# Therapeutic Plasticity of Neural Stem Cells

**DOI:** 10.3389/fneur.2020.00148

**Published:** 2020-03-20

**Authors:** Linda Ottoboni, Beatrice von Wunster, Gianvito Martino

**Affiliations:** ^1^Neurology and Neuroimmunology Unit, Institute of Experimental Neurology, San Raffaele Scientific Institute, Milan, Italy; ^2^Università Vita-Salute San Raffaele, School of Medicine, Milan, Italy

**Keywords:** neural stem cell, transplant, repair, plasticity, cell engineering

## Abstract

Neural stem cells (NSCs) have garnered significant scientific and commercial interest in the last 15 years. Given their plasticity, defined as the ability to develop into different phenotypes inside and outside of the nervous system, with a capacity of almost unlimited self-renewal, of releasing trophic and immunomodulatory factors, and of exploiting temporal and spatial dynamics, NSCs have been proposed for (i) neurotoxicity testing; (ii) cellular therapies to treat CNS diseases; (iii) neural tissue engineering and repair; (iv) drug target validation and testing; (v) personalized medicine. Moreover, given the growing interest in developing cell-based therapies to target neurodegenerative diseases, recent progress in developing NSCs from human-induced pluripotent stem cells has produced an analog of endogenous NSCs. Herein, we will review the current understanding on emerging conceptual and technological topics in the neural stem cell field, such as deep characterization of the human compartment, single-cell spatial-temporal dynamics, reprogramming from somatic cells, and NSC manipulation and monitoring. Together, these aspects contribute to further disentangling NSC plasticity to better exploit the potential of those cells, which, in the future, might offer new strategies for brain therapies.

## Introduction

The concept of the stem cell niche was officially used for the first time by Schofield (1) in 1978 to define local environments with specific molecular and cellular characteristics that are required for the maintenance of hematopoietic stem cells. Ten years previously, Smart (2) and Altman (3) identified tissue in the brain that was thought to be capable of self renewal, namely two specific regions with proliferative capacity one localized in the subventricular zone (SVZ) of the lateral ventricle and one in the subgranular zone (SGZ) of the dentate gyrus of the hippocampus. The assay to test *in vitro* neural stem/progenitor cell (NPC)-self-renewal and multi-potency consisted of assessing their ability to give rise to neurospheres (4). *In vivo*, in mice, their self-renewal capacity was proved using targeted ablation of dividing GFAP-positive cells and by genetic lineage tracing (5, 6). Similarly to the hematopoietic niche and obviously in addition to the intrinsic and specific characteristics of neural stem cells (i.e., their ability to originate neuro-glia cells), the fate of NPCs and their (lifelong) self-renewal and differentiation capacity are tightly regulated by complex interactions between intrinsic and extrinsic signals provided by surrounding cells in the niche and by distant sources (7). The microenvironment of the neurogenic niche includes multiple cell populations whose interplay, including that between stem cells themselves, is still largely unknown and under active exploration. Moreover, physical activity, stress, environmental enrichment, aging, and intrinsic factors, such as cytokines, growth factors, hormones, or neurotrophins, finely regulate the fate of neural stem cells. These features are shared with all other stem niches, such as the originally identified hematopoietic niche (8). A thorough characterization of other niche components has recently been provided in Andreotti et al. (9) and in Bacigaluppi et al. (10).

Understanding the potential of endogenous or administered NPCs as well as the cross-talk between neural stem cells and their niche components is essential for identifying what can be modulated and how for the development of therapies against neurological disorders in which neural stem cell function is altered or in which its improvement might be of help.

In this review, we would like to focus on the intrinsic and comprehensive added value of neural stem cell plasticity. NSC plasticity indeed is *per se* fundamental for development but represents an important asset in a therapeutic perspective since the neurogenic niche remains an exception in the “static” brain and represents a possible unique source of new neurons useful for substantially incurable neurological disorders and brain aging problems which are a heavy social and economic burden.

We will first frame NSCs in the stem cell context and then illustrate their plasticity in a developmental perspective, summarizing the current understanding of NSC modes of division and their mechanisms of persistence in the adult. We will compare NSCs in the two neurogenic regions of the adult mammalian mouse and human brain and discuss recent controversies on neurogenesis in the adult human brain. Last, we will discuss the current therapeutic exploitation of NSC plasticity along with the technological advancements that are being implemented, to conclude with the pros and cons, the benefits and hurdles, linked to taking advantage of these assets.

## Stem Cells

Stem cells (SC) are unspecialized, immature cells with self-renewing capacity, namely the ability to produce nearly identical copies of themselves for a long period of time without differentiating and with the possibility to differentiate into various cell lineages (11).

Totipotent stem cells, such as zygote cells and the first few cells from their division, can differentiate into all possible cell types. Pluripotent stem cells can instead differentiate into cells of the three embryonic layers, i.e., mesoderm, endoderm, and ectoderm, and can give rise to tissue and organ specialized cells. Multipotent stem cells, such as adult hematopoietic or neural stem cells, can differentiate into closely related families of cells to renew tissue-specific cell populations in organs, such as liver, intestinal tract, and skin. Exceptionally, this does not occur by default for the brain. Last, unipotent stem cells can differentiate only into a single cell type, usually of a single specialized tissue or organ.

SCs can also be classified according to their source of origin. Embryonic Stem Cells (ESCs) are totipotent, derive from the inner cell mass of human blastocysts, and can potentially proliferate indefinitely, giving rise to all types of cells in the human body. Adult Stem Cells are undifferentiated, totipotent, or multipotent cells able to replenish dying cells and to regenerate damaged tissues (if possible). Induced Pluripotent Stem Cells (iPSCs), recently developed by genetic reprogramming of adult, non-pluripotent somatic cells, are comparable to human ES cells, having differentiation potential *in vitro* and a capability to generate *in vivo* teratomas. iPSCs can be generated by over-expression through retro- or lenti-viral vector transduction of four transcription factors: Oct3/4, Sox2, c-Myc, and Klf4 [c-Myc is dispensable (12)]. These cells express human ES markers (such as OCT3/4, SOX2, and NANOG) at the same or higher level than ESCs and stain positive for markers of the three germ layers, confirming their pluripotency and differentiation potential (13). They can also be generated using small molecules that mimic the effect of transcription factors (14) or by miRNAs (15). Last, Cancer Stem Cells emerge from malignant transformation of adult stem cells or from somatic cells that acquire self-renewing potential. They have been proposed as the source of tumors and of metastases and have been isolated from various tissue types (16).

Stem cells gained value in the last 15 years for the development of cell-based therapies for many serious diseases and injuries. For, example, hematopoietic stem cell transplants became established therapeutics for leukemia and for burns and corneal disorders (11). For complex neurological diseases, unfortunately not all stem cells can be exploited. In principle, ESCs would be perfect for cell replacement therapy because they can proliferate indefinitely (17), but there is also a risk of tumor formation and immune rejection along with ethical, religious, and philosophical problems. To reduce the tumor-forming potential, human ESCs could be pre-differentiated *in vitro* in committed precursor cells or neural precursor cells (NPCs) (18), which maintain self-renewal capacity and at the same time are restricted to generate only neural cells (neurons and glia) *in vivo* upon transplant, but these still raise ethical concerns. ESCs might, in principle, be directed to differentiate into specialized neuronal subtypes (19) to further reduce the risk of tumorigenicity. But more than 200 distinct neuronal subtypes with regional specificity exist, and the applicability of transplanted differentiated cells is still far from realization.

An alternative strategy is to use neural stem/precursor cells from aborted human fetuses at the gestational age of 6–20 weeks. They can be maintained, expanded, and split without losing their self-renewing and neurogenic capacity for a long period of time *in vitro*. Their main drawback, however, is the limited availability and the unpredictability of when, where, or in what conditions the material will be obtained.

It is also possible to obtain NPCs from reprogrammed somatic cells, hiPSCs, differentiated to generate NPCs with very high neurogenic potential and virtually devoid of tumorigenicity if intracerebrally transplanted (20). Moreover, iPSC-derived NSCs, unlike adult fetal NPCs, which cannot be used as an autologous cell source, offer the possibility of autologous transplantation.

Further, NPCs could also be generated via transdifferentiation (iNPCs) of a cell type into another not following the “normal” re-programmed differentiation path, because transdifferentiated cells do not become pluripotent at any time (21–26). iNPCs, similarly to NPC-derived iPSCs, are useful for transplantation therapy, for establishing disease models, and for drug screening. In principle, they hold a low risk of tumorigenesis, maintain the capacity of self-renewal, and give rise to multiple neuronal subtypes *in vitro* and *in vivo*. Indeed, specifically for the *in vivo* applicability, murine iNPCs transplanted into healthy adult mouse brain survived for 6 months without overgrowths, achieved functional integration (27), and could differentiate into neuronal cells, although they retained a mixed neuro-glia phenotype (M2+ and GFAP+) (28). In the context of spinal cord injury, iNPCs generated by transfection with four reprogramming factors and transplanted in rat spinal cord, differentiate into all neuronal lineages (29). Direct cell conversion has also been tested *in vivo* by transplanting human fibroblasts and human astrocytes engineered to express inducible neural reprogramming genes that converted fibroblasts and astrocyte cells into neurons directly in the adult rodent brain (30).

This field is still in its infancy, and before considering the development of personalized regenerative therapies with iNPCs (31), further investigation is required to better understand the detailed mechanisms occurring in the transdifferentiation processes to improve the efficiency and the maturation into desirable cells with neurotransmitter and region-specific phenotypes.

Lastly, glial-restricted progenitor cells (GRPs) represent another therapeutic alternative. They are self-renewing cells derived from CNS tissue of 19–22 gestational weeks that have a limited differentiation potential and are able to give rise to oligodendrocytes and astrocytes but not neurons, as assessed in the demyelinated shiver mouse model (32) and in transverse myelitis, an inflammatory condition of the spinal cord that leads to demyelination (33). GRPs have also been proposed for multiple sclerosis (MS) because endogenous OPCs in the lesions initially engage in remyelination (34), but with time, the number of OPCs declines and remyelination becomes inefficient (35). Isolation and expansion of GRPs were recently implemented by Q Company (36), which started a phase I clinical trial (37).

## Neural Stem Cells in the Mammalian Brain: Fetal vs. Adult Compartment, Mouse vs. Human

**Figure 1:**
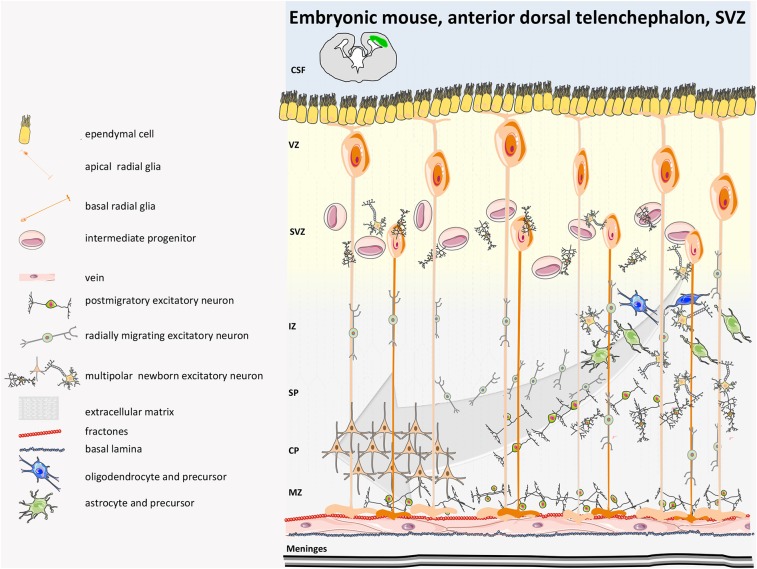
Mouse developmental SVZ structure. Neurogenesis in humans begins with the expansion of the neuroepithelium and apical radial glia (aRG). Excitatory neurons are directly generated from apical radial glia (aRG) in the dorsal VZ or are derived from multipolar basal intermediate progenitors (bIPs) that have delaminated from the apical and basal surface and reside in the SVZ. At early stages of neurogenesis in mice, newborn deep-layer excitatory neurons move basally toward the marginal zone (MZ) by somal translocation. Once the developing cortex becomes thicker, newborn neurons reach the intermediate zone (IZ), where they undergo a multipolar-to-bipolar transition and pass through the IZ and CP. Neurons then migrate basally toward the pia, passing by earlier-born neurons; they then terminate their migration in the MZ. Inhibitory GABAergic interneurons are specified in the distant medial and caudal ganglionic eminences, where RGs, intermediate progenitors (IPs), and numerous subapical progenitors (SAPs) proliferate and migrate tangentially in two streams to integrate into the various cortical layers of the cerebral cortex (not depicted in the figure). CSF, cerebrospinal fluid; SP, subplate (39–41).

**Figure 2:**
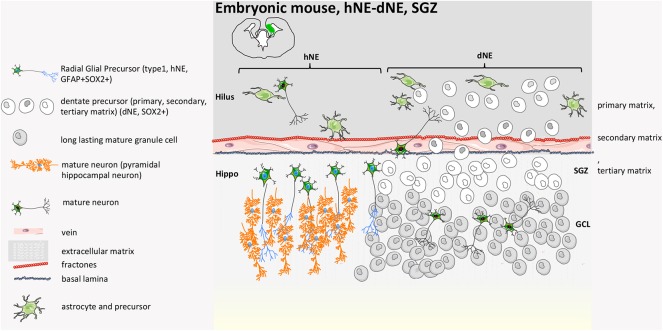
Mouse developmental SGZ structure. At E14.5, in the VZ of the hippocampal neural epithelium (hNE), radial glial precursors give rise to hippocampal pyramidal neurons. The DG originates from the dentate neuroepithelium (dNE), called the primary matrix, a part of the ventricular zone (VZ). At late gestational stages, a heterogeneous mixture of stem cells and neuronal precursors at different stages of differentiation migrate from the VZ to the hippocampal fissure, constituting a new migratory progenitor population called the secondary matrix. The process is guided by hem-derived Cajal-Retzius cells. Neural progenitors reach the hippocampal fissure, where they accumulate and form a hub of proliferating cells called the tertiary matrix (SGZ). Granule cells generated during DG development from precursors of all three matrices form the GCL. By early post-natal stages, the tertiary matrix becomes the only source of dentate progenitors and granule cells (39, 40, 42).

A detailed characterization of the neural niches for both mouse and human is now available (38). In the mouse, the central nervous system (CNS) originates at E7.5–E8 with the neural plate that folds into the neural tube and then divides along the rostro-caudal axis into the rostral forebrain, midbrain, and hindbrain vesicles, while the caudal vesicle gives rise to the spinal cord. The cortical layer, adjacent to the lateral ventricles (LV) and known as the ventricular zone (VZ) is made of highly proliferating progenitors with apical basal polarity (neuroepithelial cells, NECs) (38) that, before neurogenesis (E10.5–E12.5), undergo extensive symmetric divisions to expand (Figures 1, 2). When neurogenesis starts (E12.5 onwards), NECs become radial glial cells (RGCs), express glial markers, assume an elongated morphology, and divide asymmetrically, originating one RGC and one neuron or one RGC and one intermediate progenitor (IP, Tbr2+) (43). IPs themselves migrate radially to give rise to two pyramidal neurons that establish connection and form synapses (44). The CNS builds up in ~1 week during gestation, and NPCs (RGC/IPs) are retained in the two distinct and small proliferative areas of the SVZ and the SGZ (44, 45). During neurogenesis, first-born neurons populate the deeper cortical layers (V–VI), while later-born neurons progressively populate the more superficial layers (II–IV). These layers contain neuronal subtypes that are different in morphology, electrophysiological activity, axonal connectivity, and gene expression. During embryonic and late neurogenesis, RGCs, because of their elongated radial morphology, sense extrinsic cues from meninges, vasculature, newborn neurons, and cerebrospinal fluid, which regulate their cell fate decision. During late embryonic development and the first weeks after birth, radial glia also differentiate into astrocytes and oligodendrocytes, which populate the different brain structures, and ependymal cells will line on the ventricle surface. Thus, adult SVZ NSCs are regionally specified during the early embryonic stage and remain largely quiescent until, post-natally, they are re-activated (46, 47); they have intrinsic temporal programs linked to their positional characteristics (dorsal-ventral, rostral-caudal) (48) that guide differentiation in a cell-autonomous manner and cycle independently, but they also sense extrinsic cues that tune temporal programs and help indicate the ‘right’ time to progress (49, 50).

The functional integrity and behavior of the niche are maintained by the extracellular membranes (ECMs) (51) of both the basal and apical sides. They are rich in laminin, αβ integrin glycoproteins, and tenascin C. Similarly, a fundamental functional role for the VZ/SVZ is played by the CSF and by the blood vessels that form in early stages of CNS development (E9) (52, 53). Of note, neurogenesis and angiogenesis are regulated by the same molecules, such as vascular endothelial growth factor (VEGF), Notch, and Shh (54).

**Figure 3:**
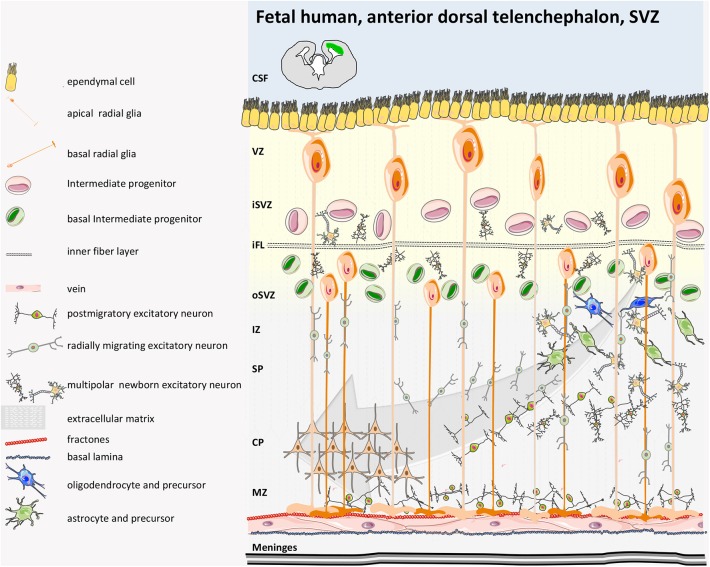
Human fetal SVZ structure. In the developing human gyrencephalic cerebral cortex, the SVZ is subdivided by the inner fiber layer (iFL) into the inner SVZ (iSVZ) and the outer SVZ (oSVZ). Neurogenesis begins with expansion of the neuroepithelium and apical radial glia (aRG) *via* asymmetrical cell cycling. Human aRGs divide to give rise to basal RG (bRGs), which delaminate from the apical surface (retaining their basal process and attachment to the pial surface), migrate basally, and populate the oSVZ. The oSVZ is also populated by basal intermediate progenitors (bIPs) that proliferate and generate neurons. The oSVZ is the predominant germinal region in the human neocortex. The basal processes of bRG act as guides for migrating newborn neurons that disperse in the tangential axis to expand the surface area of the cerebral cortex (40, 41).

**Figure 4:**
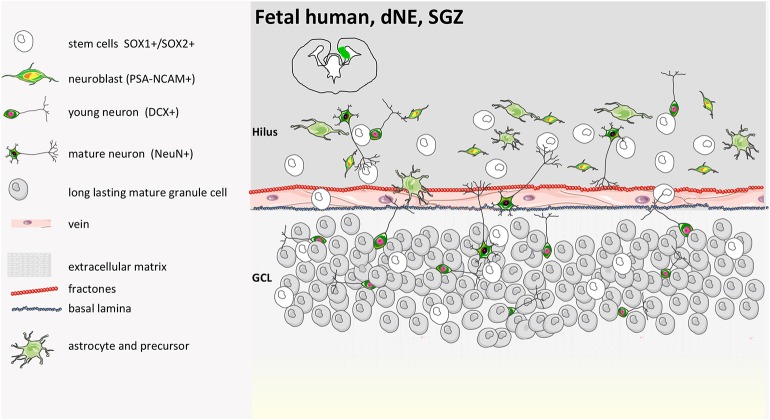
Human fetal SGZ structure. Fetal development of the SGZ starts from the dentate neuroepithelium (dNE), which is located at the edge of the ammonic neuroepithelium (aNE) close to the fimbria. SOX1+/SOX2+ precursors are organized in ribbons between dNE and GCL (granule cell layer) already at 14 gestational weeks (GW) with PSA-NCAM and DCX positive cells. SOX1 and SOX2 cells are present in the GCL and hilus and between the GCL and the dNE. A coalesced proliferative SGZ does not form in the human DG. NeuN-positive cells are seen along with SOX1 and SOX2 at 22 GW. The cellular network reported in the illustration remains until soon after birth, when either hippocampal neurogenesis continues with aging (55) or completely disappears (9, 39, 40, 55, 56).

In humans, similarly to rodents, during early brain development, the inner part of the neural tube that then becomes the cerebrospinal fluid (CSF)-filled ventricular structure consists of a layer of proliferative cells that originally contributes to the expansion of the cerebral cortex along with their descendant radial glia (GFAP+) and their intermediate progenitor cells (Figures 3, 4). Radial glia bodies are in tight contact with the monolayer of ependymal cells that covers the ventricles (57), which serves both as barrier and transport system between the interstitial fluid of the parenchyma and the CSF (58, 59). Studying the behavior of human NPCs is difficult; thus, to evaluate their properties, cells from 6.5- to 9-weeks-old aborted embryonic human forebrains were expanded in culture for up to 21 passages and were transplanted into the dentate gyrus, the rostral migratory stream (RMS), the striatum, or the SVZ of adult immunosuppressed rats. Migration was modest in the dentate gyrus or in the striatum if compared to rodent-in-rodent transplant and was considered a random dispersion process during the implantation. Larger migration was observed if transplant was into the SVZ or RMS. Cells did not show tumor formation 6 weeks post-transplantation and interestingly exclusively adopted a neuronal fate when in the olfactory bulb or in the SGZ of the hippocampus (60). It is worth mentioning also that both fetal/embryonic rodent and human NPCs display regional differences in terms of proliferation and differentiation potential according to the region of the brain where they originate (cortex or striatum) (61, 62).

In comparison to the spatially and temporally regulated niches of the developing brain, in the post-natal and adult rodent brain, neurogenesis occurs and neural stem cells (NSCs) persist in the ventricular-subventricular zone (V-SVZ) of the lateral ventricle and in the SGZ of the dentate gyrus in the hippocampus.

**Figure 5:**
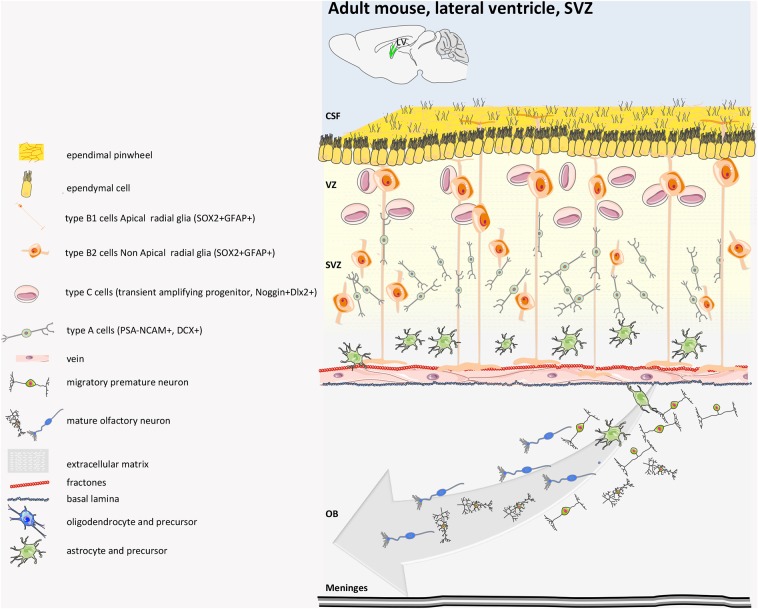
Mouse adult SVZ structure. Adult NSCs (also called radial glia-like, RGL, pre-B1 cells) of SVZ derived from embryonic radial glial (RG) cells that make neurons of the embryonic brain. The adult SVZ NSCs at embryonic day 14 (E14) upregulate p57kip2 to enter quiescence (qRGL, B1 cells). The qRGLs become activated after birth to participate in adult neurogenesis in the SVZ. In the SVZ, the RGLs mostly undergo symmetric cell division. The SVZ RGL symmetric self-renewal could occasionally also result in another type of RGL cell that lacks the apical process, named non-apical B1 cells or B2 cells. Type B1 cells give rise to neuroblast type A cells (transient amplifying cells). These young neurons are surrounded by a glial sheet and migrate anteriorly toward the olfactory bulb (OB) and differentiate in granular and periglomerular GABAergic interneurons. The adult SVZ also generates oligodendrocytes, although in much lower numbers. CSF, cerebrospinal fluid (39, 40, 63–66).

Regarding the SVZ (Figure 5), the population of adult NSCs is quite complex and heterogeneous, as demonstrated by single-cell sequencing data (67) and by marker-specific analysis (GFAP, EGFR, CD133, Nestin, CD9, CD81, CD24, and VEGF). NSCs of embryonic origin are called B1 cells (68), and there are roughly 7,000 in each young lateral wall of lateral ventricle. Most of the B1 cells generated between days E13.5 and E15.5 remain almost quiescent until soon after birth, when they become reactivated and start proliferating (46) or dividing very slowly (63, 69). NSCs divide symmetrically to self-renew or to differentiate, which leads to a decline in NSC number over time (69). B1 that face the ventricle side give rise to B2 cells, a population of fusiform-stellate proliferating V-SVZ astrocytes, that are non-neurogenic and whose function is still unknown (70). They share many astroglial characteristics with B1 cells, including contacts with blood vessels (BV), but lack contact with the apical membrane. B1 cells also generate transient-amplifying cells (type C cells) that divide symmetrically three to four times (71) and ultimately give rise to migrating neuroblasts that become young neurons (type A cells) (72). In young adult mice, B1 cells produce around 10,000 young interneurons every day that migrate for 3–8 mm along the rostral migratory stream to the olfactory bulb (73). Ventral NSCs produce deep granule cells and calbindin-positive periglomerular cells, while dorsal NSCs produce superficial granule cells and tyrosine hydroxylase-expressing periglomerular cells (74). They integrate into the existing olfactory bulb network and influence the plasticity of olfactory-related behaviors (75). B1 cells on the apical ends, which are completely surrounded by multiciliated and biciliated ependymal cells (E1 and E2 cells, respectively), which form pinwheel-like structures around them, sense the cerebrospinal fluid of the ventricle through the apical primary cilium. The choroid plexus is also considered part of the niche, and its secreted factors into the CSF regulate B1 cells. Supra-ependymal axons on the surface of the ventricular wall contact both E and B1 cells. Mature neurons and astrocytes are found below the V-SVZ (63).

The number of B1 cells drastically decreases in the first year of life in mice, but the number of newborn neurons in the olfactory bulb (OB) is not significantly affected by age, suggesting that another population of NSCs that lack apical contact and that can differentiate might exist in the adult rodent brain (69). B1 cells primarily give rise to neuro-glia cells. As regards astrocytes, B1-cell ability to differentiate into astrocytes was, for example, demonstrated upon photothrombotic ischemic cortical injury (76) and upon chemical demyelination (77). Nonetheless, it has been reported that B1 cells can also give rise to oligodendrocytes (78) destined for the corpus callosum, where they myelinate axons in both healthy (77) and demyelinating conditions (77, 79). Although dispensable in this latter condition, they protect neurons from increased axonal loss (79). Notably, post-natal and adult neurogenesis in the SVZ is carefully controlled by microglial cells (80–82).

**Figure 6:**
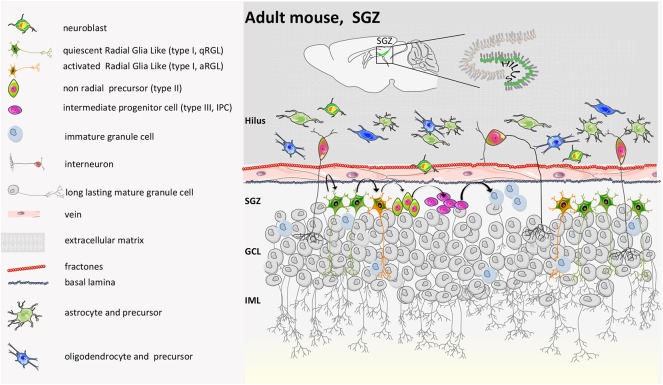
Mouse adult SGZ structure. During the second post-natal week, proliferation in the DG becomes confined to the SGZ, where NSCs reside throughout adulthood. Genetic cell lineage tracing of Sonic Hedgehog (SHH)-responsive cells has revealed that adult NSCs are induced at peri-natal stages in a restricted region next to the most ventral side of the hippocampus in close proximity to the lateral ventricle. From there, they migrate to populate all regions of the DG. Thus, embryonic and adult NSCs in the DG have different origins. Indeed, the generation of new neurons in the DG starts from radial glia-like progenitor (type I). Type I cells become activated. Activated type I cells generate intermediate progenitors (type IIa, ab, and b and type III). Type III converts into immature granule cells and finally into mature long-lasting calbindin/calretinin-positive granule cells. IML, inner molecular layer; GCL, granule cell layer. Nicola et al. showed that a condensed germinal zone in SGZ only appears during post-natal days 7–14, likely because it depends on neural activity for adult neurogenesis established by the SVZ (83). A recent report suggests that a dentate-specific neural progenitor, arising in mice at ~E11.5 and marked by *Hopx* positivity, persists from embryonic development to adulthood. These progenitors give rise at E18.5 and P7 to the dentate region and then transition to quiescence early post-natally, to contribute to neurogenesis only during the adult lifespan. Those RGLs might have limited capacity for self-renewal, are skewed toward neurogenic differentiation, and rarely make astrocytes (40, 42, 65, 84).

As regards the neural niche in the dentate gyrus (Figure 6), neurogenesis occurs on the side of the granule cell (GC) layer facing the hilum, in two or three-thin strata of the SGZ. NSCs originate in the ventral hippocampus during late gestation and then re-locate to the dorsal hippocampus. Here, quiescent NSCs, called radial glia-like (rRGL, or Type 1) cells become activated (aRGL) and divide to self-renew and to make intermediate proliferating progenitors (IPCs) that then differentiate into neuroblasts. About 25% of them survive and mature into granule neurons of the DG (85) or into mature astrocytes (86, 87) with a strategy that still needs to be fully elucidated (88–90).

While in the SVZ NSCs mainly give rise to inhibitory interneurons, in the DG, they generate new excitatory neurons that are involved in learning, memory, and pattern separation (91). New-born neurons of the SGZ are mainly located in the GC layer and do not migrate. Further, while in the SVZ, depending on the position, progenitors develop toward a different fate, the neural progenitors of the SGZ present only a bipotential fate (6). The two neurogenic niches face a 50–70% death rate during the first few days of birth, and short-living new-born neurons not only have different electrical properties than mature ones but may have their own functional role (39).

In both adult mouse neurogenic niches, it has also been reported that stem cells and differentiated daughter cells act to regulate their respective maintenance. For example, differentiated neurons release diffusible or contact-mediated signals, such as the neurotransmitter GABA and the Notch ligand Delta-like 1 (Dll1), which help to maintain NSC quiescence (92, 93). On the other hand, Tang C. and coworkers recently demonstrated a feed-forward mechanism between NSCs and newly generated neurons through pleiotrophin (PTN) ligand, whose release by NSCs supports the development of the newly differentiated neurons (94). Down the line, this cross-talk might impact the important role of striatal neurons in cognitive functions and goal-directed behavior (the dorsomedial striatum, DMS) (95), as well the sensorimotor territory and habit formation [the dorsolateral striatum (DLS)] (96).

**Figure 7:**
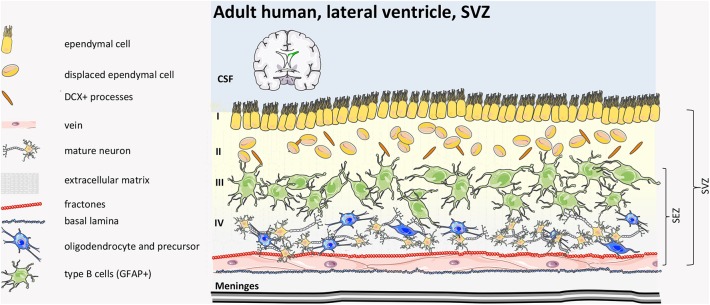
Human adult SVZ structure. The human adult SVZ consists, from the ventricle side to the parenchima, of Layer I of multicyliated ependymal cells, with radial and tangential processes, followed by a hypocellular layer (Layer II) of astrocytic and neuronal cell bodies with a number of cytoplasmic expansions of ependymal cells inserted by astrocytic ramifications. Layer III consists of a ribbon of proliferative astrocytes (type B cells). Some oligodendrocyte-like precursors and misplaced ependymal cells are found. The inner layer (Layer IV) consists primarily of myelin tracts and neuronal bodies. SEZ, sub-ependymal zone; CSF, cerebrospinal fluid (39, 40, 97).

In humans, post-natal SVZ (Figure 7) is different from in other mammals because it consists of a smaller inner and expanded outer SVZ (iSVZ and oSVZ, respectively). The oSVZ contains radial glia that support neurogenesis and cortical expansion during fetal development (98). After corticogenesis, the neurogenic niche of the iSVZ and oSVZ remains proliferative in neonates along the wall of the lateral ventricle in the site of former lateral ganglionic eminence, generating new neurons that populate the pre-frontal cortex and, partially, the olfactory bulb (81, 99) for a few months after birth. Subsequently, however, this activity declines dramatically, and, within 2 years, there is almost no detectable neurogenesis or migration (100–103). Perinatally, SVZ stem cells differentiate and migrate along three specific pathways toward the anterior forebrain: (i) to the frontal lobe where they become interneurons (arc pathway) (103); (ii) to the medial pre-frontal cortex along the medial migratory stream (MMS); (iii) to the olfactory bulb along the RMS (104). Moreover, while in many mammals newly SVZ-generated neurons migrate specifically to the olfactory bulb to guarantee olfaction throughout life (105), in the human frontal cortex, only inhibitory neurons are born post-natally with unclear function or contribute to neurocognitive maturation and plasticity, important in infancy (103, 106).

Mature, adult human SVZ consists of four layers. Moving from the ventricle side, Layer I consists of ependymal cells in contact with the lumen, and this is followed by an almost acellular layer (Layer II), which originates post-natally as a consequence of neuroblast depletion. This layer includes a dense network of astrocytes and ependymal cell processes, where astrocytes and ependymal cells exchange signaling, and the few microglial cells influence communication among the cell types (107). Adjacent to Layer II, there is a dense rim of astrocytic cell bodies (Layer III) with variable morphology. Finally, Layer IV consists of a transitional region with few cells, similar to the brain parenchyma. Although some astrocytes can proliferate (99, 108), neuroblasts are absent in the adult human SVZ niche and in the rostral migratory stream toward the olfactory bulb (101). Interestingly, in the adult human brain, newly generated cells are mainly oligodendrocytes, not neurons (109), suggesting that the oligodendrogenic process and myelin maintenance is more important in the human brain compared to other mammals.

Of note, comparing mouse and human adult SVZ, the proportion of type A:B:C cells in the mouse brain is 3:2:1, while in human, it is estimated at 1:3:1 (110).

Besides the differences between embryonic and adult niches, we have already anticipated that the niche changes during development. Indeed, a general comprehensive analysis of NPCs in mice from post-natal age P7 and P28 revealed not only that the number of NPCs decreases over the course of development but also that the genetic profile of the NPCs at the two ages was significantly different, suggesting early adulthood senescence (111).

Interestingly, it has been shown that neurons born during embryonic development (E19) and early adolescence (P21) (in mice) survived throughout adulthood (up to 2–6 months), while the cells generated at P6 displayed 15% cell death during adulthood, suggesting that early post-natal granule cells have an important unique function in terms of hippocampal plasticity (112). Early-life post-natal hippocampal neurogenesis is crucial to strengthen the ability to learn and to acquire new information via a rapid and continuous generation of new granule cells at the expense of existing memories and information storage.

Moreover, a recent report in mice showed that a population of NSCs exists in the DG that contributes to neurogenesis throughout development and adulthood and that the NSCs shift from a quiescent to an active state at different time points (84), suggesting that hippocampal neurogenesis is crucial for maintaining tissue plasticity. Indeed, the technology of single-cell RNA sequencing demonstrated that while there is an early post-natal transformation of radial glia cells from embryonic progenitors to adult quiescent stem cells maintained as such through adulthood, intermediate progenitor cells, neuroblasts, and immature granule cells are very similar at all ages (113).

**Figure 8:**
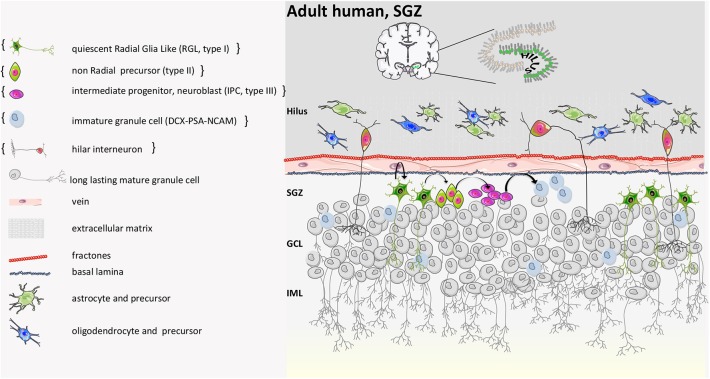
Human adult SGZ structure. Based on the report by Boldrini et al. neurogenesis persists during adulthood. The generation of new neurons starts from quiescent radial glia-like progenitor cells (type I). Type I cells become activated and then, by asymmetric division, generate intermediate progenitors (type II). Type II become neuroblasts or intermediate neural progenitors (INP type III) that convert into immature granule cells and finally mature into long-lasting granule cells that send their apical processes to the CA3 part of the hippocampus. On the other hand, according to Sorrells et al. neurogenesis is not detected in adult. In the dentate gyrus, a proliferative subgranular zone (SGZ) is not formed near the granular cell layer, and proliferative progenitor stem cells are scattered in the hilus only. They disappear anyway after 7 years from birth, and young neurons are not found in adult individuals. Curly brackets define the condition described in Sorrells et al. (9, 39, 40, 55, 56, 64, 116, 117).

Although the evidence that progenitor cells exist in the human brain is robust (114, 115) (Figure 8), controversies still persist about *in vivo* evidence that neurogenesis occurs in the adult hippocampus and about its functional relevance. A first landmark study on autoptic human brain tissue measured the concentration of ^14^C in genomic deoxyribonucleic acid (DNA) and estimated that 700 new neurons are generated each day in the adult human hippocampus corresponding to an annual turnover of 1.75% (118), comparable to what is seen in middle-aged rodents.

In this process, aging, an altered immune-related molecular and cellular status, is the most critical environmental driver that can impair neurogenesis and contribute to its decline. Indeed, when heterochronic parabiosis was attempted by exposing aged animals to a young systemic environment, adult neurogenesis increased through a-yet unknown precise mechanisms (119, 120). Moreover, Spalding et al. showed that neurogenesis also occurs in the human hippocampus in older age, in contrast to the age-related decline previously described in rodents (121). Similarly, another group observed immature and mature adult-born neurons in hippocampal post-mortem samples of healthy adult individuals (55). This evidence has recently been replicated by Moreno-Jiménez et al. who described the presence of immature neurons in the DG of 90-years-old human subjects (122) and by Tobin et al. who demonstrated that hippocampal neurogenesis occurs in the tenth decade of life (123). In contrast, a study on peri- and post-natal human samples from subjects with a wide range of diseases reported few young neurons in young individuals (7–13 years of age) and no newborn neurons in the DG of adults. Immature neurons were found only in specimens of 1-year-old subjects (56). Divergences between mouse and human are likely due to differences in the rate of generation and maturation of newborn neurons (124), while divergences among human studies might depend on limitations when using human post-mortem tissues to study neurogenesis with variable time from death to fixation.

## Features of Plasticity in Neural Stem Cells

NPCs, because of their intrinsic stem nature, exploit several plastic features. We have already mentioned their dual cell division capacity, symmetric and asymmetric, their capacity to stay quiescent in the niche for a long time and then to be activated/proliferate and to differentiate, and their regional differences, as well as their capacity to sense the environment (125) and promote neuro-biochemical changes (94). The brain is substantially a “non-renewable organ,” and brain cells slowly die with age, but neural stem cells guarantee to the CNS a certain ability to reorganize structurally and function according to intrinsic and environmental demands (126). Although proliferation and differentiation occur, it is still debated whether endogenous adult NSCs really exhibit long-term self-renewal capacity.

Encinas and collaborators reported that in the SGZ, NPCs can proliferate and then differentiate into astrocytes but that this causes progressive pauperization of the niche without returning to quiescence (127). Bonaguidi et al. instead showed, via clonal analysis, that NPCs face repetitive rounds of activation followed by astrocyte differentiation and quiescence (6). Unfortunately, different genetic labeling strategies target different populations of NSCs and different activation states of the same population, providing still complex and partial results.

Another plasticity feature for NPCs is related to the intrinsic tri-lineage potential of endogenous NSCs in the adult mammalian brain. However, although *in vitro* in culturing conditions, NSCs, both human and mouse, give rise to three different cell types, time-lapse analysis revealed generation of either neurons or oligodendrocytes, not both (128). Vice-versa, *in vivo*, different reports are available: population fate mapping has described that SVZ-NSCs generate neurons and astrocytes, not oligodendrocytes (6), while clonal analysis has recently indicated that only neuronal lineages are generated from individual NSCs (129). Cell commitment could derive from a progressive restricted lineage profile occurring in the adult (130). According to an alternative hypothesis, the trilineage potential is shaped and maintained by the niche environment (131).

The possibility that precursor cells can make new neurons is important for their therapeutic potential in neurodegenerative conditions. This potentiality has been exploited in preclinical models for stroke (132, 133). Unfortunately, after stroke, only few neuroblasts survive and differentiate, migrating from the SGZ into the granule cell layer to form novel neural circuits (134); these are not numerous enough to recover neurologic functions under ischemic conditions, and only 0.2% of lost neurons are replaced (135). Therefore, enhancing proliferation, survival, and neuronal maturation of endogenous or transplanted NSCs is important for brain disorders. Of note, while in animal models of neurodegenerative diseases like stroke, depression, epilepsy, Alzheimer's, Huntington's, and Parkinson's diseases, as well as in affected humans, progenitor cell proliferation and neurogenesis occur in the SGZ (at a lower rate in the latter and depending on the disease), in human, the SVZ is particularly sensitive to neurodegeneration and more responsive than SGZ via proliferation (136).

Under physiological conditions, the plasticity of NPCs is exploited for brain development, learning, and memory (137, 138) and is mediated by the release of trophic factors. This feature is fundamental during neurogenesis; it is important to tune synapse connections and to modulate neuronal networks during healing processes after brain injuries. Synaptic connectivity is indeed mediated by released neurotrophins (NTs), such as brain-derived neurotrophic factor (BDNF), NT-3, and NT-4 (139, 140). Depending on the type and concentration, they strengthen or weaken synaptic morphology (141) and synaptic responses (142), leading to long-term potentiation (LTP) or long-term depression (LTD). Moreover, they can form new connections and pathways and can change the wiring of the circuits (143). In severe pathological conditions, physiological trophic effects will not provide sufficient tissue regeneration. However, engraftment of exogenous stem cells, via the release of neuroprotective, trophic, or immunomodulatory factors, may stimulate endogenous neurogenesis, angiogenesis, and neovascularization, helping in the healing processes (144) and promoting the formation of new pathways around damaged tissue. The mechanism is active in the perinatal period and early-childhood, but it is progressively reduced in the brains of older children and adult individuals (145).

Cell therapy could be more beneficial when stem cells are engineered (146, 147). For example, elevated NT-3 expression can provide a microenvironment favorable to the survival and differentiation of transplanted neural stem cells (148).

For many (if not all) of the features above mentioned, epigenetic regulation plays an important role in shaping the response to the environmental cues of NPCs, depending on their developmental stage (149, 150).

The plasticity of NPCs is also exploited by their capacity to interact with scaffolds, as detailed below in the Spinal Cord Injury (SCI) section.

## Therapeutic Applications

Leveraging their plasticity, NPCs have been proposed for (i) neurotoxicity testing, (ii) cellular therapies to treat CNS conditions, (iii) neural tissue engineering and repair, (iv) drug target validation and testing, and (v) personalized medicine, as detailed below.

Since the developing CNS is more vulnerable to chemical exposure, *ad hoc* pharmacological testing is required (151). To address this goal, NSCs turned out to be useful for neurotoxicity testing. Developmental Neurotoxicity (DNT) is indeed a function not only of the type of exposure (dose, duration) but also of the developmental stage of the brain at the time of exposure (152). The blood–brain barrier (BBB) is not completely formed until at least 6 months after birth, facilitating the entrance of a chemical into the fetal/neonatal brain (153). Considering the increase of children's neurodevelopmental impairments [e.g., learning disabilities, autism, attention deficit hyperactivity disorder (ADHD)], likely due to exposures to chemicals with DNT potential, concerns have been raised about the need to identify suitable tools to properly ascertain drug toxicity. Assessment has been primarily based on animal studies, but the tests are very resource-intensive in terms of animals, time, and costs (154, 155), underlining the need to develop alternative approaches to identify DNT. *In vitro* work has been performed using rodent and human neuronal and glial cellular models (neuroblastoma cell lines) to evaluate (via dose-response relationships) the impact of a compound on various stages of brain development. Unfortunately, transformed/immortalized cell lines present limitations, such as the expression of proliferating genes that impact cellular response to chemical exposure (156, 157). On the other side, human *in vitro* neuronal cultures derived from neural progenitor cells (NPCs) or brain fetal NPCs grown as neurospheres can better mimic critical brain developmental processes, including proliferation, apoptosis, migration, and differentiation (158). However, as already mentioned, the ethical issues regulating the generation and use of human embryonic or fetal-derived tissues have been a matter of intense debate. Therefore, hiPSC-derived neuronal and glial models have been proposed for their applicability in *in vitro* pharmacological and toxicological studies. Indeed, human iPSC-derived cultures of mixed neuronal and glial cells are suitable for DNT, actually more so than for adult neurotoxic evaluation (159) because hiPSC-derived cells (and hESCs) reproduce in a more difficult way the terminal differentiation and the functional characteristics of adult brain physiology even after long term culture (160). Moreover, hiPSC-derived NPCs have an earlier neurodevelopmental phenotype because, instead of differentiating in culture into Nestin and GFAP+ like primary human NPCs, they express TubβIII (161).

Current efforts in the context of hiPSCs to optimize culturing and differentiation protocols are on-going to better mimic the brain context using defined factors and co-culture conditions. The generation of microglia-like cells from hiPSCs has helped to introduce the immune component into neuroglia culture (162–166). Further, hiPSCs have also been differentiated into brain endothelial cells that mimic the functionality of the BBB *in vitro*, adding further value to their use in DNT tests (167). Additionally, three dimensional (3D) culture and cerebral organoids have been developed and can recapitulate brain region connections occurring *in vivo* in the cerebral cortex (168). Assessing endpoints in 3D systems will be critical for guaranteeing the applicability of hiPSCs for DNT in complex assays. Combining *in vitro* DNT tests with *in vivo* epidemiological human data is crucial for developing Integrated Approaches to Testing and Assessment (IATA) for regulatory purposes (chemical screening, hazard identification/characterization, or risk assessment) (169).

NPCs surely play a crucial role in CNS tissue repair, and their intrinsic plastic nature gives them therapeutic potential in neurological diseases via two Modes of Action (MoA)L cell replacement and the bystander effect.

As regards cell replacement, NPCs are, in principle, a suitable therapeutic strategy for those diseases in which neurodegeneration and cellular loss are prevalent, not only because they mediate cell replacement but also because they can re-establish and/or support neuro-glial functional connections lost during the pathological process. This approach was tested decades ago, for example with fetal NSCs (170) transplanted into subjects with Parkinson's disease, and recently with hiPSC-derived cortical precursors transplanted into an Alzheimer's mouse model (171). However, in the first case, transplanted stem cells or progenitors could not survive for long or form the desired cell types. In the second case, human cells reproduced the pathological phenotype of AD neurons, influenced by the genetic background they had been transplanted into, suggesting that the NPC cell replacement strategy is promising only when extrinsic inflammatory neurodegenerative factors have faded in the CNS site where transplant occurs or for cell-autonomous neurological disorders. In general, transplanted NPCs give rise to the atypical ectopic perivascular niche with intense cell-to-cell cross-talk between transplanted NPCs and resident cells. NPCs may either remain in the niche in an undifferentiated state or move to acquire a terminally differentiated phenotype (172, 173) and adapt their fate according to the region of engraftment, developing neuronal or glial markers (174–176). For example, transplanted NPCs have been shown to form functional gap junctions to rescue host neurons and their projections in an animal model of Purkinje neurodegeneration (177). Moreover, human iPSC-derived NPCs have been shown to engraft and establish long-distance connections in animal models (178, 179), although concerns on the approach are still under evaluation (see below). It is crucial to mention the importance of homotopic rather than heterotopic transplantation to avoid tumorigenic risk, since region-specific cues instruct the grafts of NSCs (180). So far, this has been the predominant strategy.

Besides this evidence, NPCs might protect the CNS through mechanisms alternative to direct cell replacement, which implies the interaction of NPCs with both resident neural and immune cells. Indeed, transplanted NPCs rather exert immunomodulatory or neuroprotective functions modulating the response of the pathological processes of astrocytes, microglia, and inflammatory blood-born cells through paracrine and endocrine mechanisms (bystander effects). NPCs, upon interaction with CNS-resident cells, start releasing neurotrophic factors, such as Nerve Growth Factor-NGF, BDNF, and Glial Derived Neurotrophic Factor-GDNF, along with reactive species, binding proteins, purines, or cytokines that might significantly reduce scar formation and/or increase the survival and function of endogenous glial and neuronal progenitors. This was originally demonstrated in mice with primary inflammatory disorders, including the animal model of MS (173, 181) or stroke (182, 183) and in mice with neurodegenerative diseases mediated by reactive inflammation, such as Parkinson's Disease (184). Those properties have been then described for other stem cells, such as mesenchymal stem cells (185). The concept that therapeutic effect derives from released molecules opened the possibility of using the “secretome” of stem cells, which implies a cell-free therapeutic approach (186–191). The cross-talk with the environment is fundamental for promoting the release by NPCs of a context-specific arsenal of biological weapons, and the impact of external cues on paracrine signaling has been widely described recently (186, 192–197). However, most of the environmental cues that trigger the production of bioactive and restorative factors and the mechanisms they elicit in a specific disease are still unknown. Therefore, triggering *in vitro* the production of biologics and collecting and using the secreted factors, although promising, remains a reductionist approach, and efforts to efficiently transplant cells that sense and respond *in situ, ad hoc* to the environment are still most appropriate.

The immunomodulatory function is a feature of human NPCs (198, 199) that enables them to inhibit T-lymphocyte proliferation as well as dendritic cell maturation *in vitro*, to ameliorate disease severity when transplanted systemically in non-human primates with EAE, and to persist long-term, not only in the host CNS but also in peripheral lymph nodes (200). NPCs show pathotropism for the pathological sites, thanks to the expression of chemokine receptors, cell adhesion molecules, and integrins. Once transplanted (intravenously, i.v., or intrathecally, i.t.) and after migration into inflamed CNS areas, NPCs do no significantly differentiate but survive in close proximity to blood vessels, where they interact with CNS-infiltrating blood-derived inflammatory cells, endothelial cells, and CNS-resident astrocytes and microglia, releasing therapeutic molecules (201). In diseases characterized by primary inflammation, such as MS, stroke, or spinal cord injury, a precise control of time and route of cell administration is important to gain the therapeutic effect because NPCs transplanted in immunocompetent mice can be rejected in animals with ongoing neuroinflammation (202), and the immunomodulatory and trophic support might have a limited effect. Nonetheless, early NPC transplantation is important because, immediately after CNS damage, genes supporting tissue growth predominate over genes promoting anti-plasticity and differentiation (203).

Examples of NPC cell replacement and bystander effects for some diseases are detailed below.

### Ischemic Stroke

Stem cell transplantation for stroke has represented a valuable therapeutic strategy using various sources of NSCs. Human ESC-derived NPCs have been implanted in rodents after cerebral ischemia, and they have shown neural differentiation and improved functional recovery (204, 205). Moreover, transplanted and engrafted NSCs (i) reduced cell death and inflammation near the graft (182) and promoted angiogenesis (206); (ii) promoted proliferation and neuronal differentiation of endogenous NSCs of the subventricular and hippocampal subgranular zone in rodents (135), primates (207), and humans (208, 209); (iii) survived to intracerebral transplantation in lesioned brain and differentiated into mature neurons (178), integrating in host neuronal circuitry to promote post-stroke morphological and electrophysiological recovery (20), although several months later.

A key aspect for clinical applications of exogenous NPCs is the route of administration, which can be: (i) intraparenchymal, implying direct injection of the cell suspension close to the site of injury; this strategy achieved motor and cognitive improvements in grafted patients (210, 211), or (ii) intravascular, which is used in a limited number of trials because it is more suitable for mesenchymal stem cells. Although a greater number of cells can be administered, unfortunately, the majority does not migrate to the brain (212). Moreover, this approach retains a risk of tumorigenicity due to the possibility of heterotopic graft (180).

Since NSC transplantation in preclinical stroke models was able to promote the proliferation of endogenous NSCs and the migration of endogenous neuroblasts to the damaged brain region where they differentiate into mature neurons (213), activation of endogenous NPCs for remodeling neural tissue after ischemic injury has also been considered as a therapeutic strategy because it would not require transplantation of exogenous cells and would avoid annexed risks of introducing exogenous pathogens and of enhancing CNS immune surveillance, inflammatory reactions, and tissue rejection, as well as bypassing political and ethical concerns. In this perspective, treatment of stroke conditions with growth factors, such as epidermal growth factor (EGF) and fibroblast growth factor (FGF) promoted the recruitment of endogenous NPCs and regenerated hippocampal circuitry, restoring synaptic function after ischemia (214).

The option of using NSCs from iPSC in stroke in human is a bit more complicated to implement, due to the advanced age of most stroke patients, making it very difficult to efficiently generate iPSCs from aged subjects that can be used to perform autologous transplant. Moreover, although in some stroke models they have shown efficacy (215), it remains questionable whether iPSCs derived from aged patients are beneficial for post-stroke functional recovery (216, 217).

A phase 1 study on stroke subjects using the CTX0E03 or ReN001 cell line (ReNeuron) derived from genetically modified human fetal neuroepithelium has been conducted (211). c-mycERT AM technology was used to drive the expression of an estrogen receptor under tamoxifen (4-OHT) (in culture conditions) to control cell proliferation. Cell division was indeed arrested, and differentiation into neuronal and glial lineages was induced by removal of tamoxifen and of growth factors from the medium. Eleven men were enrolled; they did not receive any immunosuppressive therapy and were followed for 2 years. While immunological or severe adverse effects were not recorded, modest improvements on the different motor scales were observed [NIHSS, Barthel index, Ashworth Spasticity Scale for the arm and leg, and a quality-of-life and health status questionnaire, EuroQoL Five Dimensions (EQ-5D)].

### Spinal Cord Injury (SCI)

NPCs have also been intensively studied and their use proposed as a therapeutic strategy for traumatic spinal cord injury, despite the complexity of the pathology (218).

NPCs have the potential to repopulate severely injured spinal cord (197, 219), but their ability to survive and reconstitute neural tissue and neural connections remains limited by parenchyma loss and by the very toxic milieu (220). Moreover, the epicenter of the primary lesion site rapidly become necrotic, so NPCs may need an extracellular skeleton to support survival and guide tissue reorganization. Biomaterials represent a suitable support for cells, replacing the extracellular matrix to favor cell survival, differentiation, re-vascularization, and re-colonization of the tissue by glial and endothelial cells. Moreover, complex biomimetic materials that can be produced may guide axonal growth, restoring long-distance connections. More preclinical research in this innovative field is definitely required. Regenerative compounds, biomaterials, and tissue, along with cellular transplants, have been used for SCI to enhance neurite outgrowth and facilitate tissue regeneration (221). Indeed, three-dimensional highly porous “scaffolds” made of biodegradable copolymers have been tested and seeded with NPCs into the lesion to facilitate donor cell survival, migration, differentiation, functional structural repair, and neural circuit activation (222). Recently it has been reported that NPC-mediated functional recovery could depend on oligodendrocyte differentiation (223). Although NPCs have been quite extensively tested in SCI preclinical models, improvement for patients is still limited. Okano's team in Japan started a human clinical study using allogenic iPSC-derived NPCs because costs, quality testing, safety concerns, and time were not compatible with autologous transplants. Nonetheless, when immunosuppression was stopped, complications arose. Thus, so far, only autologous iPSC-derived NPCs hold promise for repair of the injured spinal cord (224).

### Neurodegenerative Diseases

NSCs may be delivered by three different routes: intravenous, intraparenchymal, or intra-cerebroventricular via lumbar puncture injection. Preclinical data have shown that via intraparenchymal delivery, NSCs migrate and spread along the corpus callosum, driven by tissue-specific disease factors (225). Via intravenous injection, NPCs cross the inflamed BBB, reaching the demyelinating areas of the CNS in animal models of multiple sclerosis (EAE) and eliciting therapeutic actions (172, 200), although NPCs could exert their bystander immunomodulatory effect also systemically. NPCs represent an effective therapeutic tool in multifocal, primary inflammatory diseases, such as multiple sclerosis, being able to migrate and exploit their bystander effect. Currently, the preclinical results in the MS context have been translated to the clinic using fetal NPCs (NCT03269071) in primary progressive MS subjects. fNPCs are currently used as a therapeutic choice also for other neurodegenerative diseases (225), and several clinical trials are in progress for neurodegenerative diseases, such as Parkinson's disease, ALS, tumors, and various pediatric diseases (not reported).

Table 1 summarizes the ongoing non-pediatric clinical trials selected on clinicaltrials.gov by using the key terms “neural stem cells,” and “neural progenitor cells.” Around 30 clinical trials reporting on transplant of NSCs have been registered on clinicaltrial.gov.

**Table 1:** List of clinical trials using NPC/NSC in adult subjects.

**Disease**	**Title**	**Trial phase**	**N^**°**^ patients**	**Age**	**Follow-up (months)**	**Cell type**	**Site and mode of administration**	**Sponsor**	**NCT Number**	**Status**
Age-Related Macular Degeneration	Study of Human Central Nervous System Stem Cells (HuCNS-SC) in Age-Related Macular Degeneration (AMD)	Phase 1 Phase 2	15	>50	12	Human neural stem cell	Subretinal space (injection)	StemCells, Inc.	NCT01632527	Completed
Amyotrophic Lateral Sclerosis	Human Neural Stem Cell Transplantation in Amyotrophic Lateral Sclerosis (hNSCALS)	Phase 1	18	20–75	36	Human fetal neural stem cell	Lumbar spinal cord (surgical device)	Azienda Ospedaliera Santa Maria, Terni, Italy	NCT01640067	Completed
Amyotrophic Lateral Sclerosis	CNS10-NPC-GDNF for the Treatment of ALS	Phase 1	18	>18	12	Human neural stem cell	Lumbar spinal cord (stereotactic device)	Cedars-Sinai Medical Center	NCT02943850	Active, not recruiting
Amyotrophic Lateral Sclerosis	Dose Escalation and Safety Study of Human Spinal Cord Derived Neural Stem Cell Transplantation for the Treatment of Amyotrophic Lateral Sclerosis	Phase 2	18	>18	24	Human neural stem cell	Spinal cord (injection)	Neuralstem Inc.	NCT01730716	Unknown status
Amyotrophic Lateral Sclerosis	Human Spinal Cord Derived Neural Stem Cell Transplantation for the Treatment of Amyotrophic Lateral Sclerosis (ALS)	Phase 1	18	>18	48	Human neural stem cell	Lumbar spinal cord (surgical implant)	Neuralstem Inc.	NCT01348451	Unknown status
Brain Tumors	Genetically Modified Neural Stem Cells, Flucytosine, and Leucovorin for Treating Patients with Recurrent High-Grade Gliomas	Phase 1	18	>18	always	Human neural stem cell	Intracranial	City of Hope Medical Center	NCT02015819	Active, not recruiting
Brain Tumors	A Pilot Feasibility Study of Oral 5-Fluorocytosine and Genetically-Modified Neural Stem Cells Expressing *E. coli* Cytosine Deaminase for Treatment of Recurrent High Grade Gliomas	Phase 1	15	>13	always	Human neural stem cell	Debulking craniotomy	City of Hope Medical Center	NCT01172964	Completed
Brain Tumors	Neural Stem Cell Based Virotherapy of Newly Diagnosed Malignant Glioma	Phase 1	36	>18	NA	Induced neural stem cells	Intracranially	Northwestern University	NCT03072134	Recruiting
Brain Tumors	Carboxylesterase-Expressing Allogeneic Neural Stem Cells and Irinotecan Hydrochloride in Treating Patients with Recurrent High-Grade Gliomas	Phase 1	53	18–69	180	Human neural stem cell	Intracranial	City of Hope Medical Center	NCT02192359	Recruiting
Ischemic Stroke	Pilot Investigation of Stem Cells in Stroke Phase II Efficacy (PISCES-II)	Phase 2	23	>40	12	Human neural stem cell	Intracerebral	ReNeuron Limited	NCT02117635	Completed
Ischemic Stroke	Intracerebral Transplantation of Neural Stem Cells for the Treatment of Ischemic Stroke	Phase 1	18	30–65	24	Human neural stem cell	Intracranial injection	Suzhou Neuralstem Biopharmaceuticals	NCT03296618	Active, not recruiting
Ischemic Stroke	Investigation of Neural Stem Cells in Ischemic Stroke (PISCES III)	Phase 2	110	35–75	12	Human neural stem cell	Stereotactic injection	ReNeuron Limited	NCT03629275	Recruiting
Ischemic Stroke	A Clinical Study of iNSC Intervent Cerebral Hemorrhagic Stroke	Early Phase 1	12	30–65	12	Induced neural stem cells	Intracerebral Transplantation	Allife Medical Science and Technology Co., Ltd.	NCT03725865	Not yet recruiting
Parkinson's Disease	A Study to Evaluate the Safety and Efficacy of Human Neural Stem Cells for Parkinson's Disease Patient (hNSCPD)	Phase 2 Phase 3	12	35–70	6	Human fetal stem cell	Nasal injection	Second Affiliated Hospital of Soochow University	NCT03128450	Unknown status
Parkinson's Disease	A Study to Evaluate the Safety of Neural Stem Cells in Patients with Parkinson's Disease	Phase 1	12	30–70	12	Induced neural stem cells	Intracerebrally to the striatum and substantia nigra	Cyto Therapeutics Pty Limited	NCT02452723	Active, not recruiting
Parkinson's Disease	A Study on the Treatment of Parkinson's Disease with Autologous Neural Stem Cells	Early Phase 1	10	18–60	12	Induced neural stem cells	NA	Allife Medical Science and Technology Co., Ltd	NCT03815071	Not yet recruiting
Parkinson's Disease	Transplantation of Neural Stem Cell-Derived Neurons for Parkinson's Disease	Phase 1 Phase 2	12	35–85	6	Human neural stem cell	Basal ganglia	NeuroGeneration	NCT03309514	Not yet recruiting
Parkinson's Disease	Safety and Efficacy Study of Human ESC-derived Neural Precursor Cells in the Treatment of Parkinson's Disease	Phase 1 Phase 2	50	50–80	12	Human embryonic stem cell-derived neural precursor cells	Intra-striatal injection	Chinese Academy of Sciences	NCT03119636	Recruiting
Pelizaeus-Merzbacher Disease (PMD)	Long-Term Follow-Up Study of Human Stem Cells Transplanted in Subjects with Connatal Pelizaeus-Merzbacher Disease (PMD)	Phase 1	4	Child, Adult, Older Adult	4	Human neural stem cell	Brain	StemCells, Inc.	NCT01391637	Completed
Peripheral Arterial Disease	Safety Trial of CTX Cells In Patients With Lower Limb Ischemia	Phase 1	5	>50	12	Human neural stem cell	Gastrocnemius muscle	ReNeuron Limited	NCT01916369	Completed
Progressive Multiple Sclerosis	Neural Stem Cell Transplantation in Multiple Sclerosis Patients (STEMS)	Phase 1	12	18–55	24	Human fetal-derived Neural Stem Cells	Intrathecal	IRCCS San Raffaele	NCT03269071	Enrolling by invitation
Secondary Progressive Multiple Sclerosis	Safety Study of Human Neural Stem Cells Injections for Secondary Progressive Multiple Sclerosis Patients (NSC-SPMS)	Phase 1	24	18–60	12	Human neural stem cell	Intraventricular	Casa Sollievo della Sofferenza IRCCS	NCT03282760	Active, not recruiting
Spinal Cord Injury	NeuroRegen Scaffold, Combined with Stem Cells for Chronic Spinal Cord Injury Repair	Phase 1 Phase 2	30	18–65	24	Human neural stem cell	Spinal cord (injection)	Chinese Academy of Sciences	NCT02688049	Enrolling by invitation
Spinal Cord Injury	Long-Term Follow-Up of Transplanted Human Central Nervous System Stem Cells (HuCNS-SC) in Spinal Cord Trauma Subjects	NA	12	18–65	NA	Human neural stem cell	Intramedullary spinal cord transplantation	StemCells, Inc.	NCT01725880	Terminated
Spinal Cord Injury	Safety Study of Human Spinal Cord-derived Neural Stem Cell Transplantation for the Treatment of Chronic SCI (SCI)	Phase 1	8	18–65	54	Human neural stem cell, spinal cord derived	N/A	Neuralstem Inc.	NCT01772810	Recruiting
Spinal Cord Injury	Study of Human Central Nervous System Stem Cells (HuCNS-SC) in Patients with Thoracic Spinal Cord Injury	Phase 1 Phase 2	12	18–60	48	Human neural stem cell	Intramedullary transplantation	StemCells, Inc.	NCT01321333	Completed

The therapeutic plasticity of NSCs has been exploited in the specific context of neural tissue engineering and repair. The development of safe techniques to generate autologous NPCs (iPSC technology and direct reprogramming of somatic cells) opened up novel therapeutic opportunities in the regenerative field. In particular, directly reprogrammed Neural Precursor Cells (drNPCs) (226) are non-immunogenic and have a stable genome and minimal risk of malignant transformation, if compared to induced-pluripotent and embryonic stem cells, while exhibiting self-renewal and multipotency.

To increase the therapeutic potential of NSCs and analogs, combination therapy of cells with engineered and miniaturized scaffolds improved spinal motor functions, as reported in a meta-analysis of more than 70 preclinical studies (227), and transplantation with tissue-engineered constructs outperformed the efficiency of suspended cells alone (228). Similarly, there has been recent testing of a “liquid matrix” strategy, which is based on platelet-rich plasma (PRP)-derived hydrogel on a solid anisotropic complex scaffold prepared using a mixture of recombinant analogs of the spider dragline silk proteins which significantly stimulated proliferation and neuronal differentiation (229). Moreover, self-assembling peptides (SAPs) have been used to generate hydrogel to support human NSC differentiation into neurons, *in vitro*, in 3D, and to test the neuroregenerative potential in rat spinal cord injuries (230). Further, graphene composites have been optimized to promote human NSC differentiation and to increase conductivity and electroactivity (231, 232), a useful strategy for peripheral nerve recovery (233).

Lastly, the therapeutic plasticity of NSCs can also be exploited in drug target validation and testing. Indeed, primary cells have the best physiological relevance, but they are limited in availability, expansion, and reproducibility, and for some diseases, they are not accessible at all. In contrast, stem cells can be propagated for a long period of time, can be cryopreserved, and can be differentiated *in vitro* into a particular lineage to model a specific disease. Moreover, research and developmental efforts have been put in place in biotech and pharmaceutical companies to generate cells for high-throughput screening (234, 235). Further, since iPSCs from patients can be differentiated into specific lineages, patient-specific derived cells have been proposed for personalized medicine. The technology is surely going to translate to the clinic for monogenic rare hereditary diseases, where iPSCs provide a model to compensate for the lack of predictive human samples or for *in-vivo* preclinical models, since CRISPR/Cas9 technology or genome manipulation can help to introduce mutations of interest (179, 236).

Moreover, the possibility of assessing the molecular consequences of drug testing at specific stages of differentiation will help to identify active pathways and possible mechanisms for target identification (237). Bioinformatics, machine-learning algorithms, and big data tools for pattern recognition can be efficiently used for data analysis, orthogonal target validation, and biomarker discovery.

## Technological Advances in the Field of NSCs That Leverage Their Therapeutic Plasticity

Recent technological advances in the field of stem cells and molecular biology have helped to potentiate their therapeutic efficacy. For example, gene therapy through the over-expression of key genes that encode for proteins with bystander potential has recently been proposed (gene therapy). This strategy has been applied to exogenous NSCs for important growth factors like NGF and BDNF. Indeed, adult human olfactory bulb neural stem/progenitor cells expressing NGF increased their proliferation and oligodendrocytic differentiation potential (238), while ESC-derived NPC expressing BDNF presented enhanced neuronal and striatal *in vivo* differentiation and turned out to be useful in Huntington's disease (239). Similarly, transplant of PSA-NCAM neural progenitors expressing BDNF was therapeutically useful in a mouse model of spinal cord injury (240), while embryonic rat NSCs expressing BDNF stimulated synaptic protein expression and promoted functional recovery in a rat model of traumatic brain injury (241). Of note, tumor formation was completely absent (242). Overexpression of GDNF was instead effective in stroke (243). The strategy has been applied not only to growth factors but also to transcription factors, such as Nurr1 (244), a critical gene in the embryonic differentiation of dopaminergic neurons (245).

Similarly, recombinant adeno-associated virus rAAVr3.45-IL10-infected human NSCs (HFT13) have been transplanted to evaluate their potential in ischemic injuries. Overexpressed IL10 had immunomodulatory effects and accelerated the recovery of neurological deficits and the reduction of brain infarction volume (246).

**Figure 9:**
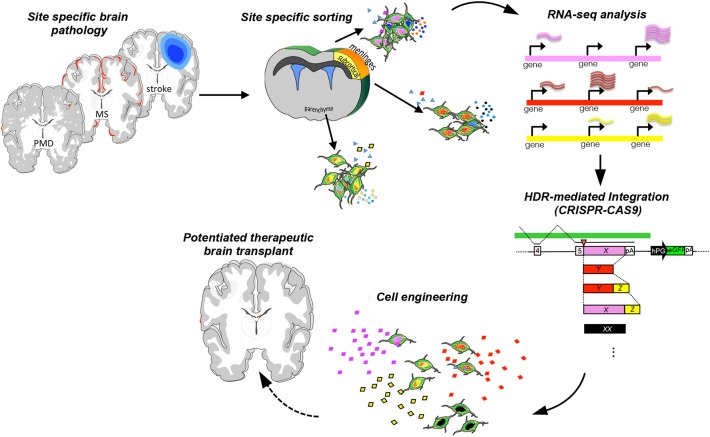
Engineering strategy to potentiate the therapeutic plasticity of neural stem cells. Transplant of NPCs in suitable preclinical neurodegenerative inflammatory and demyelinating disease models [stroke, Pelizaeus-Merzbacher disease (PMD), or multiple sclerosis], cell recovery from the pathological tissue, and then sequencing allow the identification of key molecules that exert the therapeutic effects. Further, NPCs can be engineered to potentiate *ad hoc* the expression of therapeutic targets and rescue the brain-healthy phenotype.

Engineering strategies using genome editing via CRISPR/Cas9 are being deployed on NSCs to precisely insert a gene of interest in the safe harbor human and mouse *loci* of AAVS1 and Rosa26, to perform a biallelic knockout of neurodevelopmental transcription factor genes, and to knock-in tags and fluorescent reporters (247). More recently, gene targeting at multiple *loci* using Cas9 showed great promise for a wide range of neurodegenerative disorders and injuries of the CNS, including lysosomal storage disorders (248). More sophisticated technological advances in the genome-editing field are being developed (249). Leveraging CRISPR/Cas9 for genes with ascertained therapeutic potential and with a spatio-temporal control might be possible to further harness the therapeutic plasticity of NPCs (Figure 9).

The discovery of induced pluripotency, which forces terminally differentiated adult somatic (i.e., blood or fibroblasts) cells into the pluripotent state, has provided the possibility of modeling complex neurological disorders (250). Differentiated cells are useful for screening drug candidates that can rescue molecular, cellular, and functional abnormalities in disease-specific hiPSC-derived cell types and offer the possibility of performing personalized medicine (251). In this same context, reprogramming or direct conversion of somatic cells using a non-viral system (liposome or cationic polymers) represent an interesting alternative in the perspective of clinical applicability (due to the reduced risk of tumor formation). Similarly, several types of nanoparticles useful for reprogramming have been developed. Graphene oxide-polyethylenimine complexes represent an efficient and safe system for mRNA delivery for direct reprogramming of somatic cells to induce neurons (252). Overall, on one side, the possibility of expanding *in vitro* hiPSC-derived NPCs opens up the perspective of autologous transplant and, on the other, NPCs derived from cells obtained with the new reprogramming strategy might overcome current hurdles associated with NPCs of conventional origin (both primary and from reprogrammed somatic cells) (253, 254).

It is becoming more and more important to be able to image the behavior of adult NSCs *in vivo* to explore how and where activation and division occur (255). This might be achieved with powerful microscopic and technological advances. With this aim, novel imaging sensors and tools have been developed for MRI technology, which provides excellent image quality, sensitivity, and 3D spatial resolution. Gadolinium (III) (Gd^3+^) is the heavy metal contrast agent conventionally used in clinical and animal experimental MRI. Manganese (Mn^2+^) is another useful positive T1 contrast agent that is widely used (256), similarly to iron oxide particles (SPIO), which have even higher sensitivity, better biocompatibility (function and phenotype), and increased paramagnetic power (257). Nonetheless, there are limitations in labeling stem cells with magnetic contrast agents because the label could be diluted due to stem cell proliferation after transplant. Moreover, particle loading allows stem cell tracing, but it is not informative regarding the survival state of stem cells and of possible changes induced in and by the microenvironment. Indeed, the signal could come from dead transplanted cells or cells phagocytized by microglia (258). MRI has also been improved using super-paramagnetic nanoparticles (MPI) (259) not present in biological samples, such as fluorine-19 (^19^F), a strategy that is suitable for quantification and is devoid of the ambiguity of contrast tracking (260). In addition, the resolution has been augmented by increasing the number of coil receiver channels, the strength of the magnetic field, and the number of image acquisitions.

Nuclear medicine imaging techniques, such as positron emission tomography (PET) and Single-Photon Emission Computed Tomography (SPECT), represent other promising imaging modalities for tracking stem cells. SPECT has gamma camera detectors for gamma-ray emissions from the tracers (up to two different radioisotopes at the same time) injected into the patient. PET instead measures the decay effect of different radioisotopes that emit positrons, which interact with electrons from the body, are annihilated, and generate two gamma photons emitted in opposite directions.

^111In^-oxyquinoline, ^99^mTc-HMPAO, and, mainly for the CNS, ^18^F-FDG or 2-deoxy-^18^F-FDG, 3′-deoxy-3^′18^F-FDG have been used for non-invasive imaging of NSC proliferation with PET (261, 262). It is still crucial to identify the safe dose of a radiotracer.

As an alternative to isotope cell loading and to overcome problems associated with particle loading, MRI reporter genes have been introduced for stable and robust tracking of implanted stem cells (263). The “imaging reporter genes” strategy consists of the production of a particular protein that interacts with a radioactive probe whose signal can be detected by PET/SPECT for a long time without being limited to the half-life of the tracer. With this approach, only living cells will be detected, excluding false signals (264). Cell labeling has been performed with green fluorescent protein (GFP) and red fluorescent protein (RFP), as well as with some fluorescent dyes, such as DiD, Dil, and indocyanine Green or semiconductor nanocrystals called quantum dots (QD). QDs emitting in the Near-infrared-(NIR) have been already used to track transplanted cells in the human brain (265).

Moreover, the introduction of a reporter gene that encodes for a special luciferase protein (bioluminescence imaging, BLI) has been widely applied in preclinical studies of stem cell imaging in the brain (266). Combining the high anatomical spatial resolution of MRI and the high sensitivity of PET with BLI was very useful for sensitivity and precise localization. Multimodality imaging can also be used, combining fluorescent QDs with magnetic nanoparticles (267).

Single-cell sequencing represents another fundamental technological advancement that enables the temporal and spatial dynamics of stem cells to be exploited. Since NPCs are significantly heterogeneous, each line maintained *in vitro* would need to be deeply characterized to assess the level of heterogeneity. Further, single-cell sequencing *ex vivo* on recovered transplanted cells will help develop an understanding of the therapeutic profile exploited in specific pathological conditions (Figure 9).

Obtaining data at the single-cell level helps with understanding how different types of brain cells develop and with identifying key genes to be used for cell engineering. Studies in drosophila represent an excellent model system with which to investigate how spatial and temporal factors are integrated during neurogenesis and can be translated to deep characterization in mammals (268).

## Pros and Cons of Harnessing Therapeutic Plasticity

Harnessing neural plasticity is important due to its potential to support brain healing and rewiring to fight neurological and neurodegenerative diseases and, given the physical-chemical interaction between the SVZ and the striatum, to tune neuropsychological behavior that is often associated with neurodegenerative disorders. Indeed, the neural niche represents a reservoir of cues that influence proper brain cognitive functions and decisions. An altered concentration of released soluble factors by the stem niche may be responsible for unhealthy maintenance of striatal interneurons and for modified behavioral adaptation and striatum functions, ultimately leading, in extreme conditions, to obsessive–compulsive disorders. Of note, alterations in adult neurogenesis have been linked to psychiatric disease in humans (269, 270). According to the neurogenic hypothesis, major depressive disorder (MDD) is linked to impairments of adult neurogenesis in the hippocampal DG, and antidepressants are efficacious because they increase neurogenesis (271).

Harnessing therapeutic plasticity is tantalizing, not only to balance neuronal or neurodegenerative disorders but also to open up new learning opportunities in adulthood when conventional pruning, mediated by the environmental inputs in the early phase of brain development, has already occurred and connections have already been established, through a “use it or lose it” principle. Further, this approach can be translated to aging conditions and might be useful for preserving neuronal integrity.

Plasticity is important, but the brain also needs stability. Pharmacological or genetic modification can indeed increase plasticity, but a targeted and balanced (in time and amount) intervention is fundamental because excessive release of trophic factors might be detrimental. For example, brain overexposure to TGFβ2 as an anti-inflammatory approach (272) might cause a malignant TGFβ2 autocrine loop that leads to glioblastoma (273). Excessive plasticity could also be detrimental because massive memory capabilities [Savant abilities (274)], a reflection of over-plasticity, are linked to autistic profiles, because plasticity degenerates in chaos (275). For those reasons, engineered molecular tools should be responsive to and controlled by environmental signals.

Adult NSCs reside in restricted areas of the adult CNS and have limited capacity to proliferate (276). Thus, *in vitro* expansion is a limiting factor, and growth in suspension can be troublesome. Therefore, culture in adhesion has been developed using different coatings with the ultimate goal of maintaining stable expression of stem markers, such as Nestin and Sox2. Moreover, it is always important to consider that neurospheres may be heterogeneous because they are not derived from a single NSC. On the other hand, a limited proliferation capacity might be advantageous for ensuring that NSCs do not present tumorigenic potential, and genetic stability from one passage to another is likely to be maintained.

**Figure 10:**
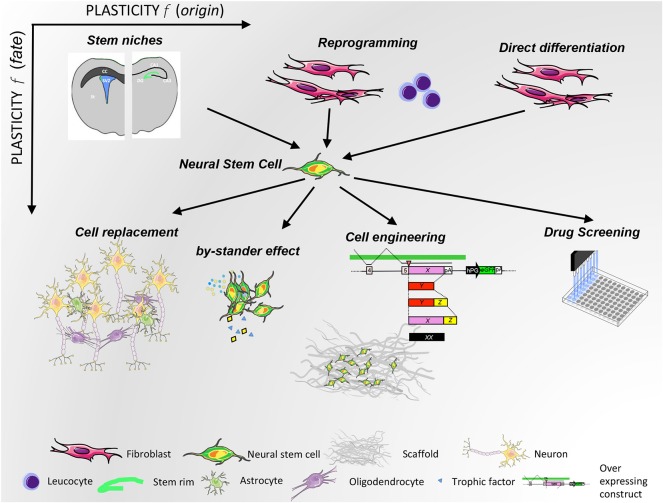
NSC plasticity as a function of origin (x-axis) and therapeutic use (y-axis). NSCs originate both from physiological niches and from *in vitro* manipulation. Their therapeutic potential is exploited with different strategies, as depicted in the lower part of the illustration.

From the perspective of expanding neural precursors in culture at large scale, the iPSC technology has helped with the generation *in vitro* of expandable and freezable samples. However, although iPSCs are an important source of NPCs, caution is necessary because of the potential risks at the genomic and epigenomic level (277). Further, NSCs derived from iPSCs could cause rejection, so they might need to be combined with an immunosuppressant. The development of non-immunogenic iPSC-based therapies is very important to minimize the probabilities of patient rejection. Nonetheless, NSCs remain the best solution for neurological diseases, compared with other stem cell types, since recovery can be promoted not only by indirect paracrine effects but also by direct neural cell replacement, which is not supported by other sources of stem cells of another developmental origin, making the latter unable to properly differentiate in the CNS (Figure 10).

## Conclusions

The discovery of neural stem cells and their potential has revived the field in terms of functional cell replacement, and concerns related to the risk of tumor formation have been dampened because the majority of NSC transplantation studies revealed no tumor formation. NSCs are a promising therapeutic approach for neurodegenerative disease. They can differentiate and replace the lost neural tissue as well as secreting neurotrophic factors that can protect or regenerate. Nonetheless, further studies are needed to quantify doses and administration periods and to define the most promising cellular NSC source considering also combined therapies to take NSCs/NPCs close to pharmacological prescription.

## Author Contributions

LO drafted the figures. All authors listed have made a substantial, direct and intellectual contribution to the work, and approved it for publication.

### Conflict of Interest

The authors declare that the research was conducted in the absence of any commercial or financial relationships that could be construed as a potential conflict of interest. The handling editor declared a past co-authorship with one of the authors, GM.

## References

[1] Schofield R. The relationship between the spleen colony-forming cell and the haemopoietic stem cell. Blood Cells. (1978) 4:7–25. 747780

[2] Smart I The subependymal layer of the mouse brain and its cell production as shown by autography after [H3]-thymidine injection. J Comp Neurol. (1961) 116:325–7. 10.1002/cne.901160306

[3] Altman J Das GD. Autoradiographic and histological evidence of postnatal hippocampal neurogenesis in rats. J Comp Neurol. (1965) 124:319–35. 10.1002/cne.901240303 5861717

[4] Reynolds BA Rietze RL. Neural stem cells and neurospheres–re-evaluating the relationship. Nat Methods. (2005) 2:333–6. 10.1038/nmeth758 15846359

[5] Garcia AD Doan NB Imura T Bush TG Sofroniew MV. GFAP-expressing progenitors are the principal source of constitutive neurogenesis in adult mouse forebrain. Nat Neurosci. (2004) 7:1233–41. 10.1038/nn1340 15494728

[6] Bonaguidi MA Wheeler MA Shapiro JS Stadel RP Sun GJ Ming GL. *In vivo* clonal analysis reveals self-renewing and multipotent adult neural stem cell characteristics. Cell. (2011) 145:1142–55. 10.1016/j.cell.2011.05.024 21664664 PMC3124562

[7] Li L Xie T. Stem cell niche: structure and function. Annu Rev Cell Dev Biol. (2005) 21:605–31. 10.1146/annurev.cellbio.21.012704.131525 16212509

[8] Leanne Jones D Fuller MT Stem Cell Niches. Essentials of Stem Cell Biology. 3rd ed. Elsevier (2014). 10.1016/C2012-0-06957-8

[9] Andreotti JP Silva WN Costa AC Picoli CC Bitencourt FCO Coimbra-Campos LMC. Neural stem cell niche heterogeneity. Semin Cell Dev Biol. (2019) 95:42–53. 10.1016/j.semcdb.2019.01.005 30639325 PMC6710163

[10] Bacigaluppi M Sferruzza G Butti E Ottoboni L Martino G. Endogenous neural precursor cells in health and disease. Brain Res. (2019) 1730:146619. 10.1016/j.brainres.2019.146619 31874148

[11] Kalra KT Tomar PC Stem cell: basics, classification and applications. AJPCT. (2014) 2:919–30.

[12] Kim JB Zaehres H Wu G Gentile L Ko K Sebastiano V. Pluripotent stem cells induced from adult neural stem cells by reprogramming with two factors. Nature. (2008) 454:646–50. 10.1038/nature07061 18594515

[13] Takahashi K Yamanaka S. Induction of pluripotent stem cells from mouse embryonic and adult fibroblast cultures by defined factors. Cell. (2006) 126:663–76. 10.1016/j.cell.2006.07.024 16904174

[14] Huangfu D Maehr R Guo W Eijkelenboom A Snitow M Chen AE. Induction of pluripotent stem cells by defined factors is greatly improved by small-molecule compounds. Nat Biotechnol. (2008) 26:795–7. 10.1038/nbt1418 18568017 PMC6334647

[15] Bao X Zhu X Liao B Benda C Zhuang Q Pei D. MicroRNAs in somatic cell reprogramming. Curr Opin Cell Biol. (2013) 25:208–14. 10.1016/j.ceb.2012.12.004 23332905

[16] Dalerba P Cho RW Clarke MF. Cancer stem cells: models and concepts. Annu Rev Med. (2007) 58:267–84. 10.1146/annurev.med.58.062105.204854 17002552

[17] Amit M Carpenter MK Inokuma MS Chiu CP Harris CP Waknitz MA. Clonally derived human embryonic stem cell lines maintain pluripotency and proliferative potential for prolonged periods of culture. Dev Biol. (2000) 227:271–8. 10.1006/dbio.2000.9912 11071754

[18] Davis CD Sanberg PR Cell Therapy, Stem Cells and Brain Repair (Contemporary Neuroscience). New Jersey, NJ: Humana Press-Totowa (2006).

[19] Batista CE Mariano ED Marie SK Teixeira MJ Morgalla M Tatagiba M. Stem cells in neurology–current perspectives. Arq Neuropsiquiatr. (2014) 72:457–65. 10.1590/0004-282X20140045 24964114

[20] Kokaia Z Darsalia V. Human neural stem cells for ischemic stroke treatment. Results Probl Cell Differ. (2018) 66:249–63. 10.1007/978-3-319-93485-3_11 30209663

[21] Han DW Tapia N Hermann A Hemmer K Hoing S Arauzo-Bravo MJ. Direct reprogramming of fibroblasts into neural stem cells by defined factors. Cell Stem Cell. (2012) 10:465–72. 10.1016/j.stem.2012.02.021 22445517

[22] Sheng C Zheng Q Wu J Xu Z Sang L Wang L. Generation of dopaminergic neurons directly from mouse fibroblasts and fibroblast-derived neural progenitors. Cell Res. (2012) 22:769–72. 10.1038/cr.2012.32 22370632 PMC3317566

[23] Lu J Liu H Huang CT Chen H Du Z Liu Y. Generation of integration-free and region-specific neural progenitors from primate fibroblasts. Cell Rep. (2013) 3:1580–91. 10.1016/j.celrep.2013.04.004 23643533 PMC3786191

[24] Cheng L Hu W Qiu B Zhao J Yu Y Guan W. Generation of neural progenitor cells by chemical cocktails and hypoxia. Cell Res. (2014) 24:665–79. 10.1038/cr.2014.32 24638034 PMC4042166

[25] Kim SM Flasskamp H Hermann A Arauzo-Bravo MJ Lee SC Lee SH. Direct conversion of mouse fibroblasts into induced neural stem cells. Nat Protoc. (2014) 9:871–81. 10.1038/nprot.2014.056 24651499

[26] Capetian P Azmitia L Pauly MG Krajka V Stengel F Bernhardi EM. Plasmid-based generation of induced neural stem cells from adult human fibroblasts. Front Cell Neurosci. (2016) 10:245. 10.3389/fncel.2016.00245 27822179 PMC5075569

[27] Hemmer K Zhang M van Wullen T Sakalem M Tapia N Baumuratov A. Induced neural stem cells achieve long-term survival and functional integration in the adult mouse brain. Stem Cell Rep. (2014) 3:423–31. 10.1016/j.stemcr.2014.06.017 25241741 PMC4265999

[28] Thier M Worsdorfer P Lakes YB Gorris R Herms S Opitz T. Direct conversion of fibroblasts into stably expandable neural stem cells. Cell Stem Cell. (2012) 10:473–9. 10.1016/j.stem.2012.03.003 22445518

[29] Hong JY Lee SH Lee SC Kim JW Kim KP Kim SM. Therapeutic potential of induced neural stem cells for spinal cord injury. J Biol Chem. (2014) 289:32512–25. 10.1074/jbc.M114.588871 25294882 PMC4239606

[30] Torper O Pfisterer U Wolf DA Pereira M Lau S Jakobsson J. Generation of induced neurons via direct conversion *in vivo*. Proc Natl Acad Sci USA. (2013) 110:7038–43. 10.1073/pnas.1303829110 23530235 PMC3637783

[31] Merrell AJ Stanger BZ. Adult cell plasticity *in vivo*: de-differentiation and transdifferentiation are back in style. Nat Rev Mol Cell Biol. (2016) 17:413–25. 10.1038/nrm.2016.24 26979497 PMC5818993

[32] Gumpel M Lachapelle F Gansmuller A Baulac M Baron van Evercooren A Baumann N. Transplantation of human embryonic oligodendrocytes into shiverer brain. Ann N Y Acad Sci. (1987) 495:71–85. 10.1111/j.1749-6632.1987.tb23666.x 3300467

[33] Walczak P All AH Rumpal N Gorelik M Kim H Maybhate A. Human glial-restricted progenitors survive, proliferate, and preserve electrophysiological function in rats with focal inflammatory spinal cord demyelination. Glia. (2011) 59:499–510. 10.1002/glia.21119 21264955 PMC3079958

[34] Chang A Nishiyama A Peterson J Prineas J Trapp BD. NG2-positive oligodendrocyte progenitor cells in adult human brain and multiple sclerosis lesions. J Neurosci. (2000) 20:6404–12. 10.1523/JNEUROSCI.20-17-06404.2000 10964946 PMC6772992

[35] Raine CS. The Norton lecture: a review of the oligodendrocyte in the multiple sclerosis lesion. J Neuroimmunol. (1997) 77:135–52. 10.1016/S0165-5728(97)00073-8 9258244

[36] Thomsen GM Gowing G Svendsen S Svendsen CN. The past, present and future of stem cell clinical trials for ALS. Exp Neurol. (2014) 262:127–37. 10.1016/j.expneurol.2014.02.021 24613827

[37] Watts JGB A phase 1/2a open-label study to investigate the safety of the transplantation (by injection) of human glial restricted progenitor cells (hGRPs; Q-cells®) into subjects with transverse myelitis (TM). Neurology. (2019) 92(Suppl. 15). Available online at: https://n.neurology.org/content/92/15_Supplement/P1.2-020

[38] Rakic P. Evolution of the neocortex: a perspective from developmental biology. Nat Rev Neurosci. (2009) 10:724–35. 10.1038/nrn2719 19763105 PMC2913577

[39] Ghosh HS. Adult neurogenesis and the promise of adult neural stem cells. J Exp Neurosci. (2019) 13:1179069519856876. 10.1177/1179069519856876 31285654 PMC6600486

[40] Morante-Redolat JM Porlan E. Neural stem cell regulation by adhesion molecules within the subependymal niche. Front Cell Dev Biol. (2019) 7:102. 10.3389/fcell.2019.00102 31245371 PMC6581678

[41] Buchsbaum IY Cappello S. Neuronal migration in the CNS during development and disease: insights from *in vivo* and *in vitro* models. Development. (2019) 146:dev163766. 10.1242/dev.163766 30626593

[42] Urban N Guillemot F. Neurogenesis in the embryonic and adult brain: same regulators, different roles. Front Cell Neurosci. (2014) 8:396. 10.3389/fncel.2014.00396 25505873 PMC4245909

[43] Paridaen JT Huttner WB. Neurogenesis during development of the vertebrate central nervous system. EMBO Rep. (2014) 15:351–64. 10.1002/embr.201438447 24639559 PMC3989667

[44] Martinez-Cerdeno V Noctor SC Kriegstein AR. The role of intermediate progenitor cells in the evolutionary expansion of the cerebral cortex. Cereb Cortex. (2006) 16:i152–61. 10.1093/cercor/bhk017 16766701

[45] Alvarez-Buylla A Garcia-Verdugo JM Tramontin AD. A unified hypothesis on the lineage of neural stem cells. Nat Rev Neurosci. (2001) 2:287–93. 10.1038/35067582 11283751

[46] Fuentealba LC Rompani SB Parraguez JI Obernier K Romero R Cepko CL. Embryonic origin of postnatal neural stem cells. Cell. (2015) 161:1644–55. 10.1016/j.cell.2015.05.041 26091041 PMC4475276

[47] Furutachi S Miya H Watanabe T Kawai H Yamasaki N Harada Y. Slowly dividing neural progenitors are an embryonic origin of adult neural stem cells. Nat Neurosci. (2015) 18:657–65. 10.1038/nn.3989 25821910

[48] Guillemot F. Cellular and molecular control of neurogenesis in the mammalian telencephalon. Curr Opin Cell Biol. (2005) 17:639–47. 10.1016/j.ceb.2005.09.006 16226447

[49] Hardwick LJ Ali FR Azzarelli R Philpott A. Cell cycle regulation of proliferation versus differentiation in the central nervous system. Cell Tissue Res. (2015) 359:187–200. 10.1007/s00441-014-1895-8 24859217 PMC4284380

[50] Yoon KJ Vissers C Ming GL Song H. Epigenetics and epitranscriptomics in temporal patterning of cortical neural progenitor competence. J Cell Biol. (2018) 217:1901–14. 10.1083/jcb.201802117 29666150 PMC5987727

[51] Sirko S von Holst A Wizenmann A Gotz M Faissner A. Chondroitin sulfate glycosaminoglycans control proliferation, radial glia cell differentiation and neurogenesis in neural stem/progenitor cells. Development. (2007) 134:2727–38. 10.1242/dev.02871 17596283

[52] Vasudevan A Long JE Crandall JE Rubenstein JL Bhide PG. Compartment-specific transcription factors orchestrate angiogenesis gradients in the embryonic brain. Nat Neurosci. (2008) 11:429–39. 10.1038/nn2074 18344991 PMC2754271

[53] Karakatsani A Shah B Ruiz de Almodovar C. Blood vessels as regulators of neural stem cell properties. Front Mol Neurosci. (2019) 12:85. 10.3389/fnmol.2019.00085 31031591 PMC6473036

[54] Carmeliet P. Angiogenesis in health and disease. Nat Med. (2003) 9:653–60. 10.1038/nm0603-653 12778163

[55] Boldrini M Fulmore CA Tartt AN Simeon LR Pavlova I Poposka V. Human hippocampal neurogenesis persists throughout aging. Cell Stem Cell. (2018) 22:589–99 e585. 10.1016/j.stem.2018.03.015 29625071 PMC5957089

[56] Sorrells SF Paredes MF Cebrian-Silla A Sandoval K Qi D Kelley KW. Human hippocampal neurogenesis drops sharply in children to undetectable levels in adults. Nature. (2018) 555:377–81. 10.1038/nature25975 29513649 PMC6179355

[57] Mirzadeh Z Merkle FT Soriano-Navarro M Garcia-Verdugo JM Alvarez-Buylla A. Neural stem cells confer unique pinwheel architecture to the ventricular surface in neurogenic regions of the adult brain. Cell Stem Cell. (2008) 3:265–78. 10.1016/j.stem.2008.07.004 18786414 PMC2613692

[58] Roales-Bujan R Paez P Guerra M Rodriguez S Vio K Ho-Plagaro A. Astrocytes acquire morphological and functional characteristics of ependymal cells following disruption of ependyma in hydrocephalus. Acta Neuropathol. (2012) 124:531–46. 10.1007/s00401-012-0992-6 22576081 PMC3444707

[59] Coletti AM Singh D Kumar S Shafin TN Briody PJ Babbitt BF. Characterization of the ventricular-subventricular stem cell niche during human brain development. Development. (2018) 145:dev170100. 10.1242/dev.170100 30237244 PMC6215394

[60] Fricker RA Carpenter MK Winkler C Greco C Gates MA Bjorklund A. Site-specific migration and neuronal differentiation of human neural progenitor cells after transplantation in the adult rat brain. J Neurosci. (1999) 19:5990–6005. 10.1523/JNEUROSCI.19-14-05990.1999 10407037 PMC6783093

[61] Ostenfeld T Joly E Tai YT Peters A Caldwell M Jauniaux E. Regional specification of rodent and human neurospheres. Brain Res Dev Brain Res. (2002) 134:43–55. 10.1016/S0165-3806(01)00291-7 11947936

[62] Martin-Ibanez R Guardia I Pardo M Herranz C Zietlow R Vinh NN. Insights in spatio-temporal characterization of human fetal neural stem cells. Exp Neurol. (2017) 291:20–35. 10.1016/j.expneurol.2017.01.011 28131724

[63] Obernier K Alvarez-Buylla A. Neural stem cells: origin, heterogeneity and regulation in the adult mammalian brain. Development. (2019) 146:dev156059. 10.1242/dev.156059 30777863 PMC6398449

[64] Zhang J Jiao J. Molecular biomarkers for embryonic and adult neural stem cell and neurogenesis. Biomed Res Int. (2015) 2015:727542. 10.1155/2015/727542 26421301 PMC4569757

[65] Shohayeb B Diab M Ahmed M Ng DCH. Factors that influence adult neurogenesis as potential therapy. Transl Neurodegener. (2018) 7:4. 10.1186/s40035-018-0109-9 29484176 PMC5822640

[66] Gonzalez-Perez O. Neural stem cells in the adult human brain. Biol Biomed Rep. (2012) 2:59–69. 10.1155/2012/378356 23181200 PMC3505091

[67] Llorens-Bobadilla E Zhao S Baser A Saiz-Castro G Zwadlo K Martin-Villalba A. Single-cell transcriptomics reveals a population of dormant neural stem cells that become activated upon brain injury. Cell Stem Cell. (2015) 17:329–40. 10.1016/j.stem.2015.07.002 26235341

[68] Doetsch F Caille I Lim DA Garcia-Verdugo JM Alvarez-Buylla A. Subventricular zone astrocytes are neural stem cells in the adult mammalian brain. Cell. (1999) 97:703–16. 10.1016/S0092-8674(00)80783-7 10380923

[69] Obernier K Cebrian-Silla A Thomson M Parraguez JI Anderson R Guinto C. Adult neurogenesis is sustained by symmetric self-renewal and differentiation. Cell Stem Cell. (2018) 22:221–34 e228. 10.1016/j.stem.2018.01.003 29395056 PMC5802882

[70] Doetsch F Garcia-Verdugo JM Alvarez-Buylla A. Cellular composition and three-dimensional organization of the subventricular germinal zone in the adult mammalian brain. J Neurosci. (1997) 17:5046–61. 10.1523/JNEUROSCI.17-13-05046.1997 9185542 PMC6573289

[71] Ponti G Obernier K Guinto C Jose L Bonfanti L Alvarez-Buylla A. Cell cycle and lineage progression of neural progenitors in the ventricular-subventricular zones of adult mice. Proc Natl Acad Sci USA. (2013) 110:E1045–54. 10.1073/pnas.1219563110 23431204 PMC3600494

[72] Lim DA Alvarez-Buylla A. The adult ventricular-subventricular zone (V-SVZ) and olfactory bulb (OB) neurogenesis. Cold Spring Harb Perspect Biol. 8:a018820. 10.1101/cshperspect.a018820 27048191 PMC4852803

[73] Bond AM Ming GL Song H. Adult mammalian neural stem cells and neurogenesis: five decades later. Cell Stem Cell. (2015) 17:385–95. 10.1016/j.stem.2015.09.003 26431181 PMC4683085

[74] Merkle FT Mirzadeh Z Alvarez-Buylla A. Mosaic organization of neural stem cells in the adult brain. Science. (2007) 317:381–4. 10.1126/science.1144914 17615304

[75] Alvarez-Buylla A Herrera DG Wichterle H. The subventricular zone: source of neuronal precursors for brain repair. Prog Brain Res. (2000) 127:1–11. 10.1016/S0079-6123(00)27002-7 11142024

[76] Benner EJ Luciano D Jo R Abdi K Paez-Gonzalez P Sheng H. Protective astrogenesis from the SVZ niche after injury is controlled by Notch modulator Thbs4. Nature. (2013) 497:369–73. 10.1038/nature12069 23615612 PMC3667629

[77] Menn B Garcia-Verdugo JM Yaschine C Gonzalez-Perez O Rowitch D Alvarez-Buylla A. Origin of oligodendrocytes in the subventricular zone of the adult brain. J Neurosci. (2006) 26:7907–18. 10.1523/JNEUROSCI.1299-06.2006 16870736 PMC6674207

[78] Nait-Oumesmar B Decker L Lachapelle F Avellana-Adalid V Bachelin C Baron-Van Evercooren A. Progenitor cells of the adult mouse subventricular zone proliferate, migrate and differentiate into oligodendrocytes after demyelination. Eur J Neurosci. (1999) 11:4357–66. 10.1046/j.1460-9568.1999.00873.x 10594662

[79] Butti E Bacigaluppi M Chaabane L Ruffini F Brambilla E Berera G. Neural stem cells of the subventricular zone contribute to neuroprotection of the corpus callosum after cuprizone-induced demyelination. J Neurosci. (2019) 39:5481–92. 10.1523/JNEUROSCI.0227-18.2019 31138656 PMC6616285

[80] Shigemoto-Mogami Y Hoshikawa K Goldman JE Sekino Y Sato K. Microglia enhance neurogenesis and oligodendrogenesis in the early postnatal subventricular zone. J Neurosci. (2014) 34:2231–43. 10.1523/JNEUROSCI.1619-13.2014 24501362 PMC3913870

[81] Matarredona ER Talaveron R Pastor AM. Interactions between neural progenitor cells and microglia in the subventricular zone: physiological implications in the neurogenic niche and after implantation in the injured brain. Front Cell Neurosci. (2018) 12:268. 10.3389/fncel.2018.00268 30177874 PMC6109750

[82] Wright-Jin EC Gutmann DH. Microglia as dynamic cellular mediators of brain function. Trends Mol Med. (2019) 25:967–79. 10.1016/j.molmed.2019.08.013 31597593 PMC6829057

[83] Nicola Z Fabel K Kempermann G. Development of the adult neurogenic niche in the hippocampus of mice. Front Neuroanat. (2015) 9:53. 10.3389/fnana.2015.00053 25999820 PMC4423450

[84] Berg DA Su Y Jimenez-Cyrus D Patel A Huang N Morizet D. A common embryonic origin of stem cells drives developmental and adult neurogenesis. Cell. (2019) 177:654–68 e615. 10.1016/j.cell.2019.02.010 30929900 PMC6496946

[85] Kempermann G Gast D Kronenberg G Yamaguchi M Gage FH. Early determination and long-term persistence of adult-generated new neurons in the hippocampus of mice. Development. (2003) 130:391–9. 10.1242/dev.00203 12466205

[86] Seri B Garcia-Verdugo JM McEwen BS Alvarez-Buylla A. Astrocytes give rise to new neurons in the adult mammalian hippocampus. J Neurosci. (2001) 21:7153–60. 10.1523/JNEUROSCI.21-18-07153.2001 11549726 PMC6762987

[87] Filippov V Kronenberg G Pivneva T Reuter K Steiner B Wang LP. Subpopulation of nestin-expressing progenitor cells in the adult murine hippocampus shows electrophysiological and morphological characteristics of astrocytes. Mol Cell Neurosci. (2003) 23:373–82. 10.1016/S1044-7431(03)00060-5 12837622

[88] Bonzano S Crisci I Podlesny-Drabiniok A Rolando C Krezel W Studer M. Neuron-astroglia cell fate decision in the adult mouse hippocampal neurogenic niche is cell-intrinsically controlled by COUP-TFI *in vivo*. Cell Rep. (2018) 24:329–41. 10.1016/j.celrep.2018.06.044 29996095

[89] Dong J Pan YB Wu XR He LN Liu XD Feng DF. A neuronal molecular switch through cell-cell contact that regulates quiescent neural stem cells. Sci Adv. (2019) 5:eaav4416. 10.1126/sciadv.aav4416 30820459 PMC6392779

[90] Wilhelmsson U Lebkuechner I Leke R Marasek P Yang X Antfolk D. Nestin regulates neurogenesis in mice through notch signaling from astrocytes to neural stem cells. Cereb Cortex. (2019) 29:4050–66. 10.1093/cercor/bhy284 30605503

[91] Ming GL Song H. Adult neurogenesis in the mammalian brain: significant answers and significant questions. Neuron. (2011) 70:687–702. 10.1016/j.neuron.2011.05.001 21609825 PMC3106107

[92] Liu X Wang Q Haydar TF Bordey A. Nonsynaptic GABA signaling in postnatal subventricular zone controls proliferation of GFAP-expressing progenitors. Nat Neurosci. (2005) 8:1179–87. 10.1038/nn1522 16116450 PMC1380263

[93] Kawaguchi D Furutachi S Kawai H Hozumi K Gotoh Y. Dll1 maintains quiescence of adult neural stem cells and segregates asymmetrically during mitosis. Nat Commun. (2013) 4:1880. 10.1038/ncomms2895 23695674 PMC3675328

[94] Tang C Wang M Wang P Wang L Wu Q Guo W. Neural stem cells behave as a functional niche for the maturation of newborn neurons through the secretion of PTN. Neuron. (2019) 101:32–44 e36. 10.1016/j.neuron.2018.10.051 30497772

[95] Redgrave P Rodriguez M Smith Y Rodriguez-Oroz MC Lehericy S Bergman H. Goal-directed and habitual control in the basal ganglia: implications for Parkinson's disease. Nat Rev Neurosci. (2010) 11:760–72. 10.1038/nrn2915 20944662 PMC3124757

[96] Graybiel AM Grafton ST. The striatum: where skills and habits meet. Cold Spring Harb Perspect Biol. (2015) 7:a021691. 10.1101/cshperspect.a021691 26238359 PMC4526748

[97] Alvarez-Palazuelos LE Robles-Cervantes MS Castillo-Velazquez G Rivas-Souza M Guzman-Muniz J Moy-Lopez N. Regulation of neural stem cell in the human SVZ by trophic and morphogenic factors. Curr Signal Transduct Ther. (2011) 6:320–6. 10.2174/157436211797483958 22053150 PMC3204663

[98] Hansen DV Lui JH Parker PR Kriegstein AR. Neurogenic radial glia in the outer subventricular zone of human neocortex. Nature. (2010) 464:554–61. 10.1038/nature08845 20154730

[99] Quinones-Hinojosa A Sanai N Soriano-Navarro M Gonzalez-Perez O Mirzadeh Z Gil-Perotin S. Cellular composition and cytoarchitecture of the adult human subventricular zone: a niche of neural stem cells. J Comp Neurol. (2006) 494:415–34. 10.1002/cne.20798 16320258

[100] Wang C Liu F Liu YY Zhao CH You Y Wang L. Identification and characterization of neuroblasts in the subventricular zone and rostral migratory stream of the adult human brain. Cell Res. (2011) 21:1534–50. 10.1038/cr.2011.83 21577236 PMC3365638

[101] Bergmann O Liebl J Bernard S Alkass K Yeung MS Steier P. The age of olfactory bulb neurons in humans. Neuron. (2012) 74:634–9. 10.1016/j.neuron.2012.03.030 22632721

[102] Villar-Cervino V Kappeler C Nobrega-Pereira S Henkemeyer M Rago L Nieto MA. Molecular mechanisms controlling the migration of striatal interneurons. J Neurosci. (2015) 35:8718–29. 10.1523/JNEUROSCI.4317-14.2015 26063906 PMC4589566

[103] Paredes MF James D Gil-Perotin S Kim H Cotter JA Ng C. Extensive migration of young neurons into the infant human frontal lobe. Science. (2016) 354:aaf7073. 10.1126/science.aaf7073 27846470 PMC5436574

[104] Sanai N Nguyen T Ihrie RA Mirzadeh Z Tsai HH Wong M. Corridors of migrating neurons in the human brain and their decline during infancy. Nature. (2011) 478:382–6. 10.1038/nature10487 21964341 PMC3197903

[105] Alunni A Bally-Cuif L. A comparative view of regenerative neurogenesis in vertebrates. Development. (2016) 143:741–53. 10.1242/dev.122796 26932669 PMC4813331

[106] Arshad A Vose LR Vinukonda G Hu F Yoshikawa K Csiszar A. Extended production of cortical interneurons into the third trimester of human gestation. Cereb Cortex. (2016) 26:2242–56. 10.1093/cercor/bhv074 25882040 PMC4830297

[107] Belenguer G Domingo-Muelas A Ferron SR Morante-Redolat JM Farinas I. Isolation, culture and analysis of adult subependymal neural stem cells. Differentiation. (2016) 91:28–41. 10.1016/j.diff.2016.01.005 27016251

[108] Capilla-Gonzalez V Herranz-Perez V Garcia-Verdugo JM. The aged brain: genesis and fate of residual progenitor cells in the subventricular zone. Front Cell Neurosci. (2015) 9:365. 10.3389/fncel.2015.00365 26441536 PMC4585225

[109] Ernst A Alkass K Bernard S Salehpour M Perl S Tisdale J. Neurogenesis in the striatum of the adult human brain. Cell. (2014) 156:1072–83. 10.1016/j.cell.2014.01.044 24561062

[110] Curtis MA Kam M Nannmark U Anderson MF Axell MZ Wikkelso C. Human neuroblasts migrate to the olfactory bulb via a lateral ventricular extension. Science. (2007) 315:1243–9. 10.1126/science.1136281 17303719

[111] Gilley JA Yang CP Kernie SG. Developmental profiling of postnatal dentate gyrus progenitors provides evidence for dynamic cell-autonomous regulation. Hippocampus. (2011) 21:33–47. 10.1002/hipo.20719 20014381 PMC2895957

[112] Ciric T Cahill SP Snyder JS. Dentate gyrus neurons that are born at the peak of development, but not before or after, die in adulthood. BioRxiv. (2019). 10.1002/brb3.1435 31576673 PMC6790299

[113] Hochgerner H Zeisel A Lonnerberg P Linnarsson S. Conserved properties of dentate gyrus neurogenesis across postnatal development revealed by single-cell RNA sequencing. Nat Neurosci. (2018) 21:290–9. 10.1038/s41593-017-0056-2 29335606

[114] Eriksson PS Perfilieva E Bjork-Eriksson T Alborn AM Nordborg C Peterson DA. Neurogenesis in the adult human hippocampus. Nat Med. (1998) 4:1313–7. 10.1038/3305 9809557

[115] Palmer TD Schwartz PH Taupin P Kaspar B Stein SA Gage FH. Cell culture. Progenitor cells from human brain after death. Nature. (2001) 411:42–3. 10.1038/35075141 11333968

[116] Eisch AJ Cameron HA Encinas JM Meltzer LA Ming GL Overstreet-Wadiche LS Adult neurogenesis, mental health, and mental illness: hope or hype? J Neurosci. (2008) 28:11785–91. 10.1523/JNEUROSCI.3798-08.2008 19005040 PMC2793333

[117] Lucassen PJ Stumpel MW Wang Q Aronica E. Decreased numbers of progenitor cells but no response to antidepressant drugs in the hippocampus of elderly depressed patients. Neuropharmacology. (2010) 58:940–9. 10.1016/j.neuropharm.2010.01.012 20138063

[118] Spalding KL Bergmann O Alkass K Bernard S Salehpour M Huttner HB. Dynamics of hippocampal neurogenesis in adult humans. Cell. (2013) 153:1219–27. 10.1016/j.cell.2013.05.002 23746839 PMC4394608

[119] Villeda SA Luo J Mosher KI Zou B Britschgi M Bieri G. The ageing systemic milieu negatively regulates neurogenesis and cognitive function. Nature. (2011) 477:90–4. 10.1038/nature10357 21886162 PMC3170097

[120] Katsimpardi L Litterman NK Schein PA Miller CM Loffredo FS Wojtkiewicz GR. Vascular and neurogenic rejuvenation of the aging mouse brain by young systemic factors. Science. (2014) 344:630–4. 10.1126/science.1251141 24797482 PMC4123747

[121] Kuhn HG Dickinson-Anson H Gage FH. Neurogenesis in the dentate gyrus of the adult rat: age-related decrease of neuronal progenitor proliferation. J Neurosci. (1996) 16:2027–33. 10.1523/JNEUROSCI.16-06-02027.1996 8604047 PMC6578509

[122] Moreno-Jimenez EP Flor-Garcia M Terreros-Roncal J Rabano A Cafini F Pallas-Bazarra N. Adult hippocampal neurogenesis is abundant in neurologically healthy subjects and drops sharply in patients with Alzheimer's disease. Nat Med. (2019) 25:554–60. 10.1038/s41591-019-0375-9 30911133

[123] Tobin MK Musaraca K Disouky A Shetti A Bheri A Honer WG. Human hippocampal neurogenesis persists in aged adults and Alzheimer's disease patients. Cell Stem Cell. (2019) 24:974–82 e973. 10.1016/j.stem.2019.05.003 31130513 PMC6608595

[124] Snyder JS. Recalibrating the relevance of adult neurogenesis. Trends Neurosci. (2019) 42:164–78. 10.1016/j.tins.2018.12.001 30686490

[125] van Praag H Kempermann G Gage FH. Neural consequences of environmental enrichment. Nat Rev Neurosci. (2000) 1:191–8. 10.1038/35044558 11257907

[126] Nitsche MA Muller-Dahlhaus F Paulus W Ziemann U. The pharmacology of neuroplasticity induced by non-invasive brain stimulation: building models for the clinical use of CNS active drugs. J Physiol. (2012) 590:4641–62. 10.1113/jphysiol.2012.232975 22869014 PMC3487028

[127] Encinas JM Michurina TV Peunova N Park JH Tordo J Peterson DA. Division-coupled astrocytic differentiation and age-related depletion of neural stem cells in the adult hippocampus. Cell Stem Cell. (2011) 8:566–79. 10.1016/j.stem.2011.03.010 21549330 PMC3286186

[128] Ortega F Gascon S Masserdotti G Deshpande A Simon C Fischer J. Oligodendrogliogenic and neurogenic adult subependymal zone neural stem cells constitute distinct lineages and exhibit differential responsiveness to Wnt signalling. Nat Cell Biol. (2013) 15:602–13. 10.1038/ncb2736 23644466

[129] Calzolari F Michel J Baumgart EV Theis F Gotz M Ninkovic J. Fast clonal expansion and limited neural stem cell self-renewal in the adult subependymal zone. Nat Neurosci. (2015) 18:490–2. 10.1038/nn.3963 25730673

[130] Kriegstein A Alvarez-Buylla A. The glial nature of embryonic and adult neural stem cells. Annu Rev Neurosci. (2009) 32:149–84. 10.1146/annurev.neuro.051508.135600 19555289 PMC3086722

[131] DeCarolis NA Mechanic M Petrik D Carlton A Ables JL Malhotra S. *in vivo* contribution of nestin- and GLAST-lineage cells to adult hippocampal neurogenesis. Hippocampus. (2013) 23:708–19. 10.1002/hipo.22130 23554226 PMC3732558

[132] Magavi SS Leavitt BR Macklis JD. Induction of neurogenesis in the neocortex of adult mice. Nature. (2000) 405:951–5. 10.1038/35016083 10879536

[133] Lindvall O Kokaia Z. Neurogenesis following stroke affecting the adult brain. Cold Spring Harb Perspect Biol. (2015) 7:a019034. 10.1101/cshperspect.a019034 26525150 PMC4632663

[134] Gage FH. Mammalian neural stem cells. Science. (2000) 287:1433–8. 10.1126/science.287.5457.1433 10688783

[135] Arvidsson A Collin T Kirik D Kokaia Z Lindvall O. Neuronal replacement from endogenous precursors in the adult brain after stroke. Nat Med. (2002) 8:963–70. 10.1038/nm747 12161747

[136] Curtis MA Low VF Faull RL. Neurogenesis and progenitor cells in the adult human brain: a comparison between hippocampal and subventricular progenitor proliferation. Dev Neurobiol. (2012) 72:990–1005. 10.1002/dneu.22028 22539366

[137] Dayan E Cohen LG. Neuroplasticity subserving motor skill learning. Neuron. (2011) 72:443–54. 10.1016/j.neuron.2011.10.008 22078504 PMC3217208

[138] Kolb B Mychasiuk R Muhammad A Gibb R. Brain plasticity in the developing brain. Prog Brain Res. (2013) 207:35–64. 10.1016/B978-0-444-63327-9.00005-9 24309250

[139] Kuczewski N Porcher C Gaiarsa JL. Activity-dependent dendritic secretion of brain-derived neurotrophic factor modulates synaptic plasticity. Eur J Neurosci. (2010) 32:1239–44. 10.1111/j.1460-9568.2010.07378.x 20880359

[140] Johansson BB. Current trends in stroke rehabilitation. A review with focus on brain plasticity. Acta Neurol Scand. (2011) 123:147–59. 10.1111/j.1600-0404.2010.01417.x 20726844

[141] Kleim JA Hogg TM VandenBerg PM Cooper NR Bruneau R Remple M Cortical synaptogenesis and motor map reorganization occur during late, but not early, phase of motor skill learning. J Neurosci. (2004) 24:628–33. 10.1523/JNEUROSCI.3440-03.2004 14736848 PMC6729261

[142] Hodgson RA Ji Z Standish S Boyd-Hodgson TE Henderson AK Racine RJ. Training-induced and electrically induced potentiation in the neocortex. Neurobiol Learn Mem. (2005) 83:22–32. 10.1016/j.nlm.2004.07.001 15607685

[143] Licht T Kreisel T Biala Y Mohan S Year Y Anisimov A. Age-dependent remarkable regenerative potential of the dentate gyrus provided by intrinsic stem cells. J Neurosci. (2020) 40:974–95. 10.1523/JNEUROSCI.1010-19.2019 31959697 PMC6989002

[144] Dihne M Hartung HP Seitz RJ. Restoring neuronal function after stroke by cell replacement: anatomic and functional considerations. Stroke. (2011) 42:2342–50. 10.1161/STROKEAHA.111.613422 21737804

[145] Merzenich MM Van Vleet TM Nahum M. Brain plasticity-based therapeutics. Front Hum Neurosci. (2014) 8:385. 10.3389/fnhum.2014.00385 25018719 PMC4072971

[146] Martinez-Serrano A Bjorklund A. Protection of the neostriatum against excitotoxic damage by neurotrophin-producing, genetically modified neural stem cells. J Neurosci. (1996) 16:4604–16. 10.1523/JNEUROSCI.16-15-04604.1996 8764649 PMC6579025

[147] Tang S Liao X Shi B Qu Y Huang Z Lin Q. The effects of controlled release of neurotrophin-3 from PCLA scaffolds on the survival and neuronal differentiation of transplanted neural stem cells in a rat spinal cord injury model. PLoS ONE. (2014) 9:e107517. 10.1371/journal.pone.0107517 25215612 PMC4162607

[148] Lu HX Hao ZM Jiao Q Xie WL Zhang JF Lu YF. Neurotrophin-3 gene transduction of mouse neural stem cells promotes proliferation and neuronal differentiation in organotypic hippocampal slice cultures. Med Sci Monit. (2011) 17:BR305–11. 10.12659/MSM.882039 22037732 PMC3539508

[149] Urayama S Semi K Sanosaka T Hori Y Namihira M Kohyama J. Chromatin accessibility at a STAT3 target site is altered prior to astrocyte differentiation. Cell Struct Funct. (2013) 38:55–66. 10.1247/csf.12034 23439558

[150] Douvaras P Rusielewicz T Kim KH Haines JD Casaccia P Fossati V. Epigenetic modulation of human induced pluripotent stem cell differentiation to oligodendrocytes. Int J Mol Sci. (2016) 17:614. 10.3390/ijms17040614 27110779 PMC4849063

[151] Grandjean P Landrigan PJ. Developmental neurotoxicity of industrial chemicals. Lancet. (2006) 368:2167–78. 10.1016/S0140-6736(06)69665-7 17174709

[152] Rice D Barone S Jr. Critical periods of vulnerability for the developing nervous system: evidence from humans and animal models. Environ Health Perspect. (2000) 108:511–33. 10.1289/ehp.00108s3511 10852851 PMC1637807

[153] Rodier PM. Developing brain as a target of toxicity. Environ Health Perspect. (1995) 103:73–6. 10.1289/ehp.95103s673 8549496 PMC1518932

[154] Tsuji R Crofton KM. Developmental neurotoxicity guideline study: issues with methodology, evaluation and regulation. Congenit Anom (Kyoto). (2012) 52:122–8. 10.1111/j.1741-4520.2012.00374.x 22925212

[155] Bal-Price A Hogberg HT Crofton KM Daneshian M FitzGerald RE Fritsche E. Recommendation on test readiness criteria for new approach methods in toxicology: exemplified for developmental neurotoxicity. ALTEX. (2018) 35:306–52. 10.14573/altex.1712081 29485663 PMC6545888

[156] Bal-Price AK Coecke S Costa L Crofton KM Fritsche E Goldberg A. Advancing the science of developmental neurotoxicity (DNT): testing for better safety evaluation. ALTEX. (2012) 29:202–15. 10.14573/altex.2012.2.202 22892558

[157] Bal-Price A Pistollato F Sachana M Bopp SK Munn S Worth A. Strategies to improve the regulatory assessment of developmental neurotoxicity (DNT) using *in vitro* methods. Toxicol Appl Pharmacol. (2018) 354:7–18. 10.1016/j.taap.2018.02.008 29476865 PMC6095942

[158] Schmuck MR Temme T Dach K de Boer D Barenys M Bendt F. Omnisphero: a high-content image analysis (HCA) approach for phenotypic developmental neurotoxicity (DNT) screenings of organoid neurosphere cultures *in vitro*. Arch Toxicol. (2017) 91:2017–28. 10.1007/s00204-016-1852-2 27722930

[159] Pistollato F Canovas-Jorda D Zagoura D Price A Protocol for the differentiation of human induced pluripotent stem cells into mixed cultures of neurons and glia for neurotoxicity testing. J Vis Exp. (2017) 124:e55702 10.3791/55702 PMC5608344 28654077

[160] Amin H Maccione A Marinaro F Zordan S Nieus T Berdondini L. Electrical responses and spontaneous activity of human iPS-derived neuronal networks characterized for 3-month culture with 4096-electrode arrays. Front Neurosci. (2016) 10:121. 10.3389/fnins.2016.00121 27065786 PMC4811967

[161] Hofrichter M Nimtz L Tigges J Kabiri Y Schroter F Royer-Pokora B. Comparative performance analysis of human iPSC-derived and primary neural progenitor cells (NPC) grown as neurospheres *in vitro*. Stem Cell Res. (2017) 25:72–82. 10.1016/j.scr.2017.10.013 29112887

[162] Abud EM Ramirez RN Martinez ES Healy LM Nguyen CHH Newman SA. iPSC-derived human microglia-like cells to study neurological diseases. Neuron. (2017) 94:278–93 e279. 10.1016/j.neuron.2017.03.042 28426964 PMC5482419

[163] Haenseler W Sansom SN Buchrieser J Newey SE Moore CS Nicholls FJ. A highly efficient human pluripotent stem cell microglia model displays a neuronal-co-culture-specific expression profile and inflammatory response. Stem Cell Rep. (2017) 8:1727–42. 10.1016/j.stemcr.2017.05.017 28591653 PMC5470330

[164] Pandya H Shen MJ Ichikawa DM Sedlock AB Choi Y Johnson KR. Differentiation of human and murine induced pluripotent stem cells to microglia-like cells. Nat Neurosci. (2017) 20:753–9. 10.1038/nn.4534 28253233 PMC5404968

[165] McQuade A Coburn M Tu CH Hasselmann J Davtyan H Blurton-Jones M. Development and validation of a simplified method to generate human microglia from pluripotent stem cells. Mol Neurodegener. (2018) 13:67. 10.1186/s13024-018-0297-x 30577865 PMC6303871

[166] Muffat J Li Y Omer A Durbin A Bosch I Bakiasi G. Human induced pluripotent stem cell-derived glial cells and neural progenitors display divergent responses to Zika and dengue infections. Proc Natl Acad Sci USA. (2018) 115:7117–22. 10.1073/pnas.1719266115 29915057 PMC6142255

[167] Canfield SG Stebbins MJ Faubion MG Gastfriend BD Palecek SP Shusta EV An isogenic neurovascular unit model comprised of human induced pluripotent stem cell-derived brain microvascular endothelial cells, pericytes, astrocytes, and neurons. Fluids Barriers CNS. (2019) 16:25 10.1186/s12987-019-0151-8 31387594 PMC6685239

[168] Lancaster MA Renner M Martin CA Wenzel D Bicknell LS Hurles ME. Cerebral organoids model human brain development and microcephaly. Nature. (2013) 501:373–9. 10.1038/nature12517 23995685 PMC3817409

[169] Baumann J Gassmann K Masjosthusmann S DeBoer D Bendt F Giersiefer S. Comparative human and rat neurospheres reveal species differences in chemical effects on neurodevelopmental key events. Arch Toxicol. (2016) 90:1415–27. 10.1007/s00204-015-1568-8 26216354

[170] Madrazo I Leon V Torres C Aguilera MC Varela G Alvarez F. Transplantation of fetal substantia nigra and adrenal medulla to the caudate nucleus in two patients with Parkinson's disease. N Engl J Med. (1988) 318:51. 10.1056/NEJM198801073180115 3336384

[171] Espuny-Camacho I Arranz AM Fiers M Snellinx A Ando K Munck S. Hallmarks of Alzheimer's disease in stem-cell-derived human neurons transplanted into mouse brain. Neuron. (2017) 93:1066–81 e1068. 10.1016/j.neuron.2017.02.001 28238547

[172] Pluchino S Quattrini A Brambilla E Gritti A Salani G Dina G. Injection of adult neurospheres induces recovery in a chronic model of multiple sclerosis. Nature. (2003) 422:688–94. 10.1038/nature01552 12700753

[173] Pluchino S Zanotti L Deleidi M Martino G. Neural stem cells and their use as therapeutic tool in neurological disorders. Brain Res Brain Res Rev. (2005) 48:211–9. 10.1016/j.brainresrev.2004.12.011 15850660

[174] Chu K Kim M Park KI Jeong SW Park HK Jung KH. Human neural stem cells improve sensorimotor deficits in the adult rat brain with experimental focal ischemia. Brain Res. (2004) 1016:145–53. 10.1016/j.brainres.2004.04.038 15246850

[175] Takeuchi H Natsume A Wakabayashi T Aoshima C Shimato S Ito M. Intravenously transplanted human neural stem cells migrate to the injured spinal cord in adult mice in an SDF-1- and HGF-dependent manner. Neurosci Lett. (2007) 426:69–74. 10.1016/j.neulet.2007.08.048 17884290

[176] Sinden JD Hicks C Stroemer P Vishnubhatla I Corteling R. Human neural stem cell therapy for chronic ischemic stroke: charting progress from laboratory to patients. Stem Cells Dev. (2017) 26:933–47. 10.1089/scd.2017.0009 28446071 PMC5510676

[177] Jaderstad J Jaderstad LM Li J Chintawar S Salto C Pandolfo M. Communication via gap junctions underlies early functional and beneficial interactions between grafted neural stem cells and the host. Proc Natl Acad Sci USA. (2010) 107:5184–9. 10.1073/pnas.0915134107 20147621 PMC2841882

[178] Tornero D Wattananit S Gronning Madsen M Koch P Wood J Tatarishvili J. Human induced pluripotent stem cell-derived cortical neurons integrate in stroke-injured cortex and improve functional recovery. Brain. (2013) 136:3561–77. 10.1093/brain/awt278 24148272

[179] Thompson LH Bjorklund A. Reconstruction of brain circuitry by neural transplants generated from pluripotent stem cells. Neurobiol Dis. (2015) 79:28–40. 10.1016/j.nbd.2015.04.003 25913029

[180] Melzi R Antonioli B Mercalli A Battaglia M Valle A Pluchino S. Co-graft of allogeneic immune regulatory neural stem cells (NPC) and pancreatic islets mediates tolerance, while inducing NPC-derived tumors in mice. PLoS ONE. (2010) 5:e10357. 10.1371/journal.pone.0010357 20436918 PMC2860511

[181] Martino G Pluchino S. The therapeutic potential of neural stem cells. Nat Rev Neurosci. (2006) 7:395–406. 10.1038/nrn1908 16760919

[182] Bacigaluppi M Pluchino S Peruzzotti-Jametti L Kilic E Kilic U Salani G. Delayed post-ischaemic neuroprotection following systemic neural stem cell transplantation involves multiple mechanisms. Brain. (2009) 132:2239–51. 10.1093/brain/awp174 19617198

[183] Baker EW Kinder HA West FD. Neural stem cell therapy for stroke: a multimechanistic approach to restoring neurological function. Brain Behav. (2019) 9:e01214. 10.1002/brb3.1214 30747485 PMC6422715

[184] Richardson RM Broaddus WC Holloway KL Fillmore HL. Grafts of adult subependymal zone neuronal progenitor cells rescue hemiparkinsonian behavioral decline. Brain Res. (2005) 1032:11–22. 10.1016/j.brainres.2004.10.043 15680936

[185] Ottoboni L De Feo D Merlini A Martino G. Commonalities in immune modulation between mesenchymal stem cells (MSCs) and neural stem/precursor cells (NPCs). Immunol Lett. (2015) 168:228–39. 10.1016/j.imlet.2015.05.005 25986012

[186] Drago D Cossetti C Iraci N Gaude E Musco G Bachi A. The stem cell secretome and its role in brain repair. Biochimie. (2013) 95:2271–85. 10.1016/j.biochi.2013.06.020 23827856 PMC4061727

[187] Pluchino S Cossetti C. How stem cells speak with host immune cells in inflammatory brain diseases. Glia. (2013) 61:1379–401. 10.1002/glia.22500 23633288 PMC4063195

[188] Sutaria DS Badawi M Phelps MA Schmittgen TD. Achieving the promise of therapeutic extracellular vesicles: the devil is in details of therapeutic loading. Pharm Res. (2017) 34:1053–66. 10.1007/s11095-017-2123-5 28315083 PMC5565485

[189] Mendes-Pinheiro B Teixeira FG Anjo SI Manadas B Behie LA Salgado AJ. Secretome of undifferentiated neural progenitor cells induces histological and motor improvements in a rat model of Parkinson's disease. Stem Cells Transl Med. (2018) 7:829–38. 10.1002/sctm.18-0009 30238668 PMC6216452

[190] Yang H Wang C Chen H Li L Ma S Wang H. Neural stem cell-conditioned medium ameliorated cerebral ischemia-reperfusion injury in rats. Stem Cells Int. (2018) 2018:4659159. 10.1155/2018/4659159 29765412 PMC5903322

[191] Teixeira FG Salgado AJ. Mesenchymal stem cells secretome: current trends and future challenges. Neural Regen Res. (2020) 15:75–7. 10.4103/1673-5374.264455 31535654 PMC6862404

[192] Cossetti C Iraci N Mercer TR Leonardi T Alpi E Drago D. Extracellular vesicles from neural stem cells transfer IFN-gamma via Ifngr1 to activate Stat1 signaling in target cells. Mol Cell. (2014) 56:193–204. 10.1016/j.molcel.2014.08.020 25242146 PMC4578249

[193] Madhavan L Daley BF Davidson BL Boudreau RL Lipton JW Cole-Strauss A. Sonic hedgehog controls the phenotypic fate and therapeutic efficacy of grafted neural precursor cells in a model of nigrostriatal neurodegeneration. PLoS ONE. (2015) 10:e0137136. 10.1371/journal.pone.0137136 26340267 PMC4560385

[194] Di Santo S Widmer HR. Paracrine factors for neurodegenerative disorders: special emphasis on Parkinson's disease. Neural Regen Res. (2016) 11:570–1. 10.4103/1673-5374.180739 27212915 PMC4870911

[195] Webb RL Kaiser EE Jurgielewicz BJ Spellicy S Scoville SL Thompson TA. Human neural stem cell extracellular vesicles improve recovery in a porcine model of ischemic stroke. Stroke. (2018) 49:1248–56. 10.1161/STROKEAHA.117.020353 29650593 PMC5916046

[196] Webb RL Kaiser EE Scoville SL Thompson TA Fatima S Pandya C. Human neural stem cell extracellular vesicles improve tissue and functional recovery in the murine thromboembolic stroke model. Transl Stroke Res. (2018) 9:530–9. 10.1007/s12975-017-0599-2 29285679 PMC6132936

[197] Yuan T Liu Q Kang J Gao H Gui S. High-dose neural stem/progenitor cell transplantation increases engraftment and neuronal distribution and promotes functional recovery in rats after acutely severe spinal cord injury. Stem Cells Int. (2019) 2019:9807978. 10.1155/2019/9807978 31565061 PMC6745168

[198] Fainstein N Cohen ME Ben-Hur T. Time associated decline in neurotrophic properties of neural stem cell grafts render them dependent on brain region-specific environmental support. Neurobiol Dis. (2013) 49:41–8. 10.1016/j.nbd.2012.08.004 22910454

[199] Fainstein N Einstein O Cohen ME Brill L Lavon I Ben-Hur T. Time limited immunomodulatory functions of transplanted neural precursor cells. Glia. (2013) 61:140–9. 10.1002/glia.22420 23001547

[200] Pluchino S Gritti A Blezer E Amadio S Brambilla E Borsellino G. Human neural stem cells ameliorate autoimmune encephalomyelitis in non-human primates. Ann Neurol. (2009) 66:343–54. 10.1002/ana.21745 19798728

[201] De Feo D Merlini A Laterza C Martino G. Neural stem cell transplantation in central nervous system disorders: from cell replacement to neuroprotection. Curr Opin Neurol. (2012) 25:322–33. 10.1097/WCO.0b013e328352ec45 22547103

[202] Weinger JG Weist BM Plaisted WC Klaus SM Walsh CM Lane TE. MHC mismatch results in neural progenitor cell rejection following spinal cord transplantation in a model of viral-induced demyelination. Stem Cells. (2012) 30:2584–95. 10.1002/stem.1234 22969049 PMC3479361

[203] Darsalia V Allison SJ Cusulin C Monni E Kuzdas D Kallur T. Cell number and timing of transplantation determine survival of human neural stem cell grafts in stroke-damaged rat brain. J Cereb Blood Flow Metab. (2011) 31:235–42. 10.1038/jcbfm.2010.81 20531461 PMC3049487

[204] Daadi MM Davis AS Arac A Li Z Maag AL Bhatnagar R. Human neural stem cell grafts modify microglial response and enhance axonal sprouting in neonatal hypoxic-ischemic brain injury. Stroke. (2010) 41:516–23. 10.1161/STROKEAHA.109.573691 20075340 PMC5512869

[205] Jiang P Chen C Wang R Chechneva OV Chung SH Rao MS. hESC-derived Olig2+ progenitors generate a subtype of astroglia with protective effects against ischaemic brain injury. Nat Commun. (2013) 4:2196. 10.1038/ncomms3196 23880652 PMC3903179

[206] Zhang P Li J Liu Y Chen X Lu H Kang Q. Human embryonic neural stem cell transplantation increases subventricular zone cell proliferation and promotes peri-infarct angiogenesis after focal cerebral ischemia. Neuropathology. (2011) 31:384–91. 10.1111/j.1440-1789.2010.01182.x 21175862

[207] Tonchev AB Yamashima T Zhao L Okano H. Differential proliferative response in the postischemic hippocampus, temporal cortex, and olfactory bulb of young adult macaque monkeys. Glia. (2003) 42:209–24. 10.1002/glia.10209 12673828

[208] Nakayama D Matsuyama T Ishibashi-Ueda H Nakagomi T Kasahara Y Hirose H. Injury-induced neural stem/progenitor cells in post-stroke human cerebral cortex. Eur J Neurosci. (2010) 31:90–8. 10.1111/j.1460-9568.2009.07043.x 20104652

[209] Hao L Zou Z Tian H Zhang Y Zhou H Liu L. Stem cell-based therapies for ischemic stroke. Biomed Res Int. (2014) 2014:468748. 10.1155/2014/468748 24719869 PMC3955655

[210] Kondziolka D Steinberg GK Wechsler L Meltzer CC Elder E Gebel J. Neurotransplantation for patients with subcortical motor stroke: a phase 2 randomized trial. J Neurosurg. (2005) 103:38–45. 10.3171/jns.2005.103.1.0038 16121971

[211] Kalladka D Sinden J Pollock K Haig C McLean J Smith W. Human neural stem cells in patients with chronic ischaemic stroke (PISCES): a phase 1, first-in-man study. Lancet. (2016) 388:787–96. 10.1016/S0140-6736(16)30513-X 27497862

[212] Lappalainen RS Narkilahti S Huhtala T Liimatainen T Suuronen T Narvanen A. The SPECT imaging shows the accumulation of neural progenitor cells into internal organs after systemic administration in middle cerebral artery occlusion rats. Neurosci Lett. (2008) 440:246–50. 10.1016/j.neulet.2008.05.090 18572314

[213] Mine Y Tatarishvili J Oki K Monni E Kokaia Z Lindvall O. Grafted human neural stem cells enhance several steps of endogenous neurogenesis and improve behavioral recovery after middle cerebral artery occlusion in rats. Neurobiol Dis. (2013) 52:191–203. 10.1016/j.nbd.2012.12.006 23276704

[214] Nakatomi H Kuriu T Okabe S Yamamoto S Hatano O Kawahara N. Regeneration of hippocampal pyramidal neurons after ischemic brain injury by recruitment of endogenous neural progenitors. Cell. (2002) 110:429–41. 10.1016/S0092-8674(02)00862-0 12202033

[215] Oki K Tatarishvili J Wood J Koch P Wattananit S Mine Y. Human-induced pluripotent stem cells form functional neurons and improve recovery after grafting in stroke-damaged brain. Stem Cells. (2012) 30:1120–33. 10.1002/stem.1104 22495829

[216] Kokaia Z Llorente IL Carmichael ST. Customized brain cells for stroke patients using pluripotent stem cells. Stroke. (2018) 49:1091–8. 10.1161/STROKEAHA.117.018291 29669871 PMC5916498

[217] Strassler ET Aalto-Setala K Kiamehr M Landmesser U Krankel N. Age is relative-impact of donor age on induced pluripotent stem cell-derived cell functionality. Front Cardiovasc Med. (2018) 5:4. 10.3389/fcvm.2018.00004 29423397 PMC5790033

[218] Badner A Siddiqui AM Fehlings MG. Spinal cord injuries: how could cell therapy help? Expert Opin Biol Ther. (2017) 17:529–41. 10.1080/14712598.2017.1308481 28306359

[219] Cusimano M Biziato D Brambilla E Donega M Alfaro-Cervello C Snider S. Transplanted neural stem/precursor cells instruct phagocytes and reduce secondary tissue damage in the injured spinal cord. Brain. (2012) 135:447–60. 10.1093/brain/awr339 22271661 PMC3558737

[220] Cummings BJ Uchida N Tamaki SJ Salazar DL Hooshmand M Summers R. Human neural stem cells differentiate and promote locomotor recovery in spinal cord-injured mice. Proc Natl Acad Sci USA. (2005) 102:14069–74. 10.1073/pnas.0507063102 16172374 PMC1216836

[221] Thompson R Sakiyama-Elbert S. Using biomaterials to promote pro-regenerative glial phenotypes after nervous system injuries. Biomed Mater. (2018) 13:024104. 10.1088/1748-605X/aa9e23 29186011 PMC5825184

[222] Teng YD Wang L Zeng X Wu L Toktas Z Kabatas S. Updates on human neural stem cells: from generation, maintenance, and differentiation to applications in spinal cord injury research. Results Probl Cell Differ. (2018) 66:233–48. 10.1007/978-3-319-93485-3_10 30209662

[223] Sankavaram SR Hakim R Covacu R Frostell A Neumann S Svensson M. Adult neural progenitor cells transplanted into spinal cord injury differentiate into oligodendrocytes, enhance myelination, and contribute to recovery. Stem Cell Rep. (2019) 12:950–66. 10.1016/j.stemcr.2019.03.013 31031190 PMC6524946

[224] Pereira IM Marote A Salgado AJ Silva NA. Filling the gap: neural stem cells as a promising therapy for spinal cord injury. Pharmaceuticals (Basel). (2019) 12:E65. 10.3390/ph12020065 31035689 PMC6631328

[225] Ferrari D Gelati M Profico DC Vescovi AL. Human fetal neural stem cells for neurodegenerative disease treatment. Results Probl Cell Differ. (2018) 66:307–29. 10.1007/978-3-319-93485-3_14 30209666

[226] Nagoshi N Khazaei M Ahlfors JE Ahuja CS Nori S Wang J. Human spinal oligodendrogenic neural progenitor cells promote functional recovery after spinal cord injury by axonal remyelination and tissue sparing. Stem Cells Transl Med. (2018) 7:806–18. 10.1002/sctm.17-0269 30085415 PMC6216444

[227] Romanyuk N Amemori T Turnovcova K Prochazka P Onteniente B Sykova E. Beneficial effect of human induced pluripotent stem cell-derived neural precursors in spinal cord injury repair. Cell Transplant. (2015) 24:1781–97. 10.3727/096368914X684042 25259685

[228] Zweckberger K Ahuja CS Liu Y Wang J Fehlings MG. Self-assembling peptides optimize the post-traumatic milieu and synergistically enhance the effects of neural stem cell therapy after cervical spinal cord injury. Acta Biomater. (2016) 42:77–89. 10.1016/j.actbio.2016.06.016 27296842

[229] Baklaushev VP Bogush VG Kalsin VA Sovetnikov NN Samoilova EM Revkova VA. Tissue engineered neural constructs composed of neural precursor cells, recombinant spidroin and PRP for neural tissue regeneration. Sci Rep. (2019) 9:3161. 10.1038/s41598-019-39341-9 30816182 PMC6395623

[230] Marchini A Raspa A Pugliese R El Malek MA Pastori V Lecchi M. Multifunctionalized hydrogels foster hNSC maturation in 3D cultures and neural regeneration in spinal cord injuries. Proc Natl Acad Sci USA. (2019) 116:7483–92. 10.1073/pnas.1818392116 30923117 PMC6462084

[231] Solanki A Chueng ST Yin PT Kappera R Chhowalla M Lee KB. Axonal alignment and enhanced neuronal differentiation of neural stem cells on graphene-nanoparticle hybrid structures. Adv Mater. (2013) 25:5477–82. 10.1002/adma.201302219 23824715 PMC4189106

[232] Jakus AE Secor EB Rutz AL Jordan SW Hersam MC Shah RN. Three-dimensional printing of high-content graphene scaffolds for electronic and biomedical applications. ACS Nano. (2015) 9:4636–48. 10.1021/acsnano.5b01179 25858670

[233] Bei HP Yang Y Zhang Q Tian Y Luo X Yang M. Graphene-based nanocomposites for neural tissue engineering. Molecules. (2019) 24:E658. 10.3390/molecules24040658 30781759 PMC6413135

[234] Avior Y Sagi I Benvenisty N. Pluripotent stem cells in disease modelling and drug discovery. Nat Rev Mol Cell Biol. (2016) 17:170–82. 10.1038/nrm.2015.27 26818440

[235] Engle SJ Blaha L Kleiman RJ. Best practices for translational disease modeling using human iPSC-derived neurons. Neuron. (2018) 100:783–97. 10.1016/j.neuron.2018.10.033 30465765

[236] Lyu C Shen J Wang R Gu H Zhang J Xue F. Targeted genome engineering in human induced pluripotent stem cells from patients with hemophilia B using the CRISPR-Cas9 system. Stem Cell Res Ther. (2018) 9:92. 10.1186/s13287-018-0839-8 29625575 PMC5889534

[237] Kusumoto D Yuasa S. The application of convolutional neural network to stem cell biology. Inflamm Regen. (2019) 39:14. 10.1186/s41232-019-0103-3 31312276 PMC6611022

[238] Marei HE Althani A Afifi N Abd-Elmaksoud A Bernardini C Michetti F. Over-expression of hNGF in adult human olfactory bulb neural stem cells promotes cell growth and oligodendrocytic differentiation. PLoS ONE. (2013) 8:e82206. 10.1371/journal.pone.0082206 24367504 PMC3868548

[239] Zimmermann T Remmers F Lutz B Leschik J. ESC-derived BDNF-overexpressing neural progenitors differentially promote recovery in Huntington's disease models by enhanced striatal differentiation. Stem Cell Rep. (2016) 7:693–706. 10.1016/j.stemcr.2016.08.018 27693427 PMC5063570

[240] Butenschon J Zimmermann T Schmarowski N Nitsch R Fackelmeier B Friedemann K. PSA-NCAM positive neural progenitors stably expressing BDNF promote functional recovery in a mouse model of spinal cord injury. Stem Cell Res Ther. (2016) 7:11. 10.1186/s13287-015-0268-x 26762640 PMC4712602

[241] Ma H Yu B Kong L Zhang Y Shi Y. Neural stem cells over-expressing brain-derived neurotrophic factor (BDNF) stimulate synaptic protein expression and promote functional recovery following transplantation in rat model of traumatic brain injury. Neurochem Res. (2012) 37:69–83. 10.1007/s11064-011-0584-1 21901549

[242] Aubry L Bugi A Lefort N Rousseau F Peschanski M Perrier AL. Striatal progenitors derived from human ES cells mature into DARPP32 neurons *in vitro* and in quinolinic acid-lesioned rats. Proc Natl Acad Sci USA. (2008) 105:16707–12. 10.1073/pnas.0808488105 18922775 PMC2575484

[243] Takahashi K Yasuhara T Shingo T Muraoka K Kameda M Takeuchi A. Embryonic neural stem cells transplanted in middle cerebral artery occlusion model of rats demonstrated potent therapeutic effects, compared to adult neural stem cells. Brain Res. (2008) 1234:172–82. 10.1016/j.brainres.2008.07.086 18703033

[244] Shim JW Park CH Bae YC Bae JY Chung S Chang MY. Generation of functional dopamine neurons from neural precursor cells isolated from the subventricular zone and white matter of the adult rat brain using Nurr1 overexpression. Stem Cells. (2007) 25:1252–62. 10.1634/stemcells.2006-0274 17234994

[245] Wagner J Akerud P Castro DS Holm PC Canals JM Snyder EY. Induction of a midbrain dopaminergic phenotype in Nurr1-overexpressing neural stem cells by type 1 astrocytes. Nat Biotechnol. (1999) 17:653–9. 10.1038/10862 10404157

[246] Cho M Jung K Kim SH Kim IS Kim M Shin M. Safety and efficacy evaluations of an adeno-associated virus variant for preparing IL10-secreting human neural stem cell-based therapeutics. Gene Ther. (2019) 26:135–50. 10.1038/s41434-019-0057-8 30692604

[247] Bressan RB Dewari PS Kalantzaki M Gangoso E Matjusaitis M Garcia-Diaz C. Efficient CRISPR/Cas9-assisted gene targeting enables rapid and precise genetic manipulation of mammalian neural stem cells. Development. (2017) 144:635–48. 10.1242/dev.140855 28096221 PMC5312033

[248] Dever DP Scharenberg SG Camarena J Kildebeck EJ Clark JT Martin RM. CRISPR/Cas9 genome engineering in engraftable human brain-derived neural stem cells. iScience. (2019) 15:524–35. 10.1016/j.isci.2019.04.036 31132746 PMC6538928

[249] Anzalone AV Randolph PB Davis JR Sousa AA Koblan LW Levy JM Search-and-replace genome editing without double-strand breaks or donor DNA. Nature. (2019) 576:149–57. 10.1038/s41586-019-1711-4 31634902 PMC6907074

[250] Takahashi K Tanabe K Ohnuki M Narita M Ichisaka T Tomoda K. Induction of pluripotent stem cells from adult human fibroblasts by defined factors. Cell. (2007) 131:861–72. 10.1016/j.cell.2007.11.019 18035408

[251] Zhang J Li H Trounson A Wu JC Nioi P. Combining hiPSCs and human genetics: major applications in drug development. Cell Stem Cell. (2017) 21:161–5. 10.1016/j.stem.2017.07.012 28777942

[252] Baek S Oh J Song J Choi H Yoo J Park GY. Generation of integration-free induced neurons using graphene oxide-polyethylenimine. Small. (2017) 13:16019993. 10.1002/smll.201601993 28145631

[253] Ramos-Zuniga R Gonzalez-Perez O Macias-Ornelas A Capilla-Gonzalez V Quinones-Hinojosa A. Ethical implications in the use of embryonic and adult neural stem cells. Stem Cells Int. (2012) 2012:470949. 10.1155/2012/470949 22997522 PMC3444931

[254] Popp B Krumbiegel M Grosch J Sommer A Uebe S Kohl Z. Need for high-resolution genetic analysis in iPSC: results and lessons from the ForIPS consortium. Sci Rep. (2018) 8:17201. 10.1038/s41598-018-35506-0 30464253 PMC6249203

[255] Manganas LN Zhang X Li Y Hazel RD Smith SD Wagshul ME. Magnetic resonance spectroscopy identifies neural progenitor cells in the live human brain. Science. (2007) 318:980–5. 10.1126/science.1147851 17991865 PMC4039561

[256] Pan D Schmieder AH Wickline SA Lanza GM. Manganese-based MRI contrast agents: past, present and future. Tetrahedron. (2011) 67:8431–44. 10.1016/j.tet.2011.07.076 22043109 PMC3203535

[257] Them K Salamon J Szwargulski P Sequeira S Kaul MG Lange C. Increasing the sensitivity for stem cell monitoring in system-function based magnetic particle imaging. Phys Med Biol. (2016) 61:3279–90. 10.1088/0031-9155/61/9/3279 27032447

[258] Liu W Frank JA. Detection and quantification of magnetically labeled cells by cellular MRI. Eur J Radiol. (2009) 70:258–64. 10.1016/j.ejrad.2008.09.021 18995978 PMC2680943

[259] Du Y Lai PT Leung CH Pong PW. Design of superparamagnetic nanoparticles for magnetic particle imaging (MPI). Int J Mol Sci. (2013) 14:18682–710. 10.3390/ijms140918682 24030719 PMC3794803

[260] Srinivas M Boehm-Sturm P Aswendt M Pracht ED Figdor CG de Vries IJ *in vivo* 19F MRI for cell tracking. J Vis Exp. (2013) e50802. 10.3791/50802 PMC3992025 24299964

[261] Gleave JA Valliant JF Doering LC. 99mTc-based imaging of transplanted neural stem cells and progenitor cells. J Nucl Med Technol. (2011) 39:114–20. 10.2967/jnmt.111.087445 21565954

[262] Rueger MA Ameli M Li H Winkeler A Rueckriem B Vollmar S. [18F]FLT PET for non-invasive monitoring of early response to gene therapy in experimental gliomas. Mol Imaging Biol. (2011) 13:547–57. 10.1007/s11307-010-0361-6 20563754

[263] Cromer Berman SM Walczak P Bulte JW. Tracking stem cells using magnetic nanoparticles. Wiley Interdiscip Rev Nanomed Nanobiotechnol. (2011) 3:343–55. 10.1002/wnan.140 21472999 PMC3193153

[264] Qin C Cheng K Chen K Hu X Liu Y Lan X. Tyrosinase as a multifunctional reporter gene for Photoacoustic/MRI/PET triple modality molecular imaging. Sci Rep. (2013) 3:1490. 10.1038/srep01490 23508226 PMC3603217

[265] Chen G Tian F Li C Zhang Y Weng Z Zhang Y. *in vivo* real-time visualization of mesenchymal stem cells tropism for cutaneous regeneration using NIR-II fluorescence imaging. Biomaterials. (2015) 53:265–73. 10.1016/j.biomaterials.2015.02.090 25890725

[266] Kruttwig K Brueggemann C Kaijzel E Vorhagen S Hilger T Lowik C. Development of a three-dimensional *in vitro* model for longitudinal observation of cell behavior: monitoring by magnetic resonance imaging and optical imaging. Mol Imaging Biol. (2010) 12:367–76. 10.1007/s11307-009-0289-x 19949979 PMC2912723

[267] Koole R Mulder WJ van Schooneveld MM Strijkers GJ Meijerink A Nicolay K. Magnetic quantum dots for multimodal imaging. Wiley Interdiscip Rev Nanomed Nanobiotechnol. (2009) 1:475–91. 10.1002/wnan.14 20049812

[268] Sen SQ Chanchani S Southall TD Doe CQ. Neuroblast-specific open chromatin allows the temporal transcription factor, Hunchback, to bind neuroblast-specific loci. Elife. (2019) 8:e44036. 10.7554/eLife.44036.026 30694180 PMC6377230

[269] Eisch AJ Petrik D. Depression and hippocampal neurogenesis: a road to remission? Science. (2012) 338:72–5. 10.1126/science.1222941 23042885 PMC3756889

[270] Kheirbek MA Klemenhagen KC Sahay A Hen R. Neurogenesis and generalization: a new approach to stratify and treat anxiety disorders. Nat Neurosci. (2012) 15:1613–20. 10.1038/nn.3262 23187693 PMC3638121

[271] Tunc-Ozcan E Peng CY Zhu Y Dunlop SR Contractor A Kessler JA. Activating newborn neurons suppresses depression and anxiety-like behaviors. Nat Commun. (2019) 10:3768. 10.1038/s41467-019-11641-8 31434877 PMC6704083

[272] De Feo D Merlini A Brambilla E Ottoboni L Laterza C Menon R. Neural precursor cell-secreted TGF-beta2 redirects inflammatory monocyte-derived cells in CNS autoimmunity. J Clin Invest. (2017) 127:3937–53. 10.1172/JCI92387 28945200 PMC5663358

[273] Rodon L Gonzalez-Junca A Inda Mdel M Sala-Hojman A Martinez-Saez E Seoane J. Active CREB1 promotes a malignant TGFbeta2 autocrine loop in glioblastoma. Cancer Discov. (2014) 4:1230–41. 10.1158/2159-8290.CD-14-0275 25084773

[274] Treffert DA. The savant syndrome: an extraordinary condition. A synopsis: past, present, future. Philos Trans R Soc Lond B Biol Sci. (2009) 364:1351–7. 10.1098/rstb.2008.0326 19528017 PMC2677584

[275] Chen JA Penagarikano O Belgard TG Swarup V Geschwind DH. The emerging picture of autism spectrum disorder: genetics and pathology. Annu Rev Pathol. (2015) 10:111–44. 10.1146/annurev-pathol-012414-040405 25621659

[276] Signer RA Morrison SJ. Mechanisms that regulate stem cell aging and life span. Cell Stem Cell. (2013) 12:152–65. 10.1016/j.stem.2013.01.001 23395443 PMC3641677

[277] Guhr A Kobold S Seltmann S Seiler Wulczyn AEM Kurtz A Loser P. Recent trends in research with human pluripotent stem cells: impact of research and use of cell lines in experimental research and clinical trials. Stem Cell Rep. (2018) 11:485–96. 10.1016/j.stemcr.2018.06.012 30033087 PMC6092712

